# SNPmanifold: detecting single-cell clonality and lineages from single-nucleotide variants using binomial variational autoencoder

**DOI:** 10.1186/s13059-025-03803-3

**Published:** 2025-09-26

**Authors:** Hoi Man Chung, Yuanhua Huang

**Affiliations:** 1 School of Biomedical Sciences, University of Hong Kong, Hong Kong SAR, China; 2 Department of Statistics and Actuarial Science, University of Hong Kong, Hong Kong SAR, China

## Abstract

Single-nucleotide-variant (SNV) clone assignment of high-covariance single-cell lineage tracing data remains a challenge due to hierarchical mutation structure and many missing signals. We develop SNPmanifold, a Python package that learns an SNV embedding manifold using a binomial variational autoencoder to give an efficient and interpretable cell-cell distance metric. We demonstrate that SNPmanifold is a suitable tool for analysis of complex, single-cell SNV mutation data, such as in the context of demultiplexing a large number of donors and somatic lineage tracing via mitochondrial SNV data and can reveal insights into single-cell clonality and lineages more accurately and comprehensively than existing methods.

**Supplementary Information:**

The online version contains supplementary material available at 10.1186/s13059-025-03803-3.

## Background

Single-cell genomics is the study of mutations in DNA sequences of individual cells using omics approaches. It has various applications [1, 2] in oncology, prenatal diagnosis, tissue mosaicism, immunology, organogenesis, embryogenesis, germline transmission, microbiology, and neurobiology, where single-cell genetic heterogeneity (e.g., SNVs; single nucleotide variants) is prominent and informative either as markers [3] or pleiotropic mutations [4]. To investigate single-cell genetic heterogeneity, researchers developed various sequencing protocols [5–9] and associated statistical methods to analyze such data. For SNVs (single nucleotide variants), existing analysis methods fall into two main types (Table 1): (1) one-by-one SNP (single nucleotide polymorphism) selection methods which use *p*-value (or other probability metrics) ranking, and (2) genotype-demultiplexing methods which use low-dimensional mixture models with constraints. In other words, one may characterize cells with the selected individual SNPs one at a time or with the estimated clonal labels together with the inferred genotype vectors. Of note, one may combine the type 1 and 2 methods by selecting informative variants first and then performing a (clonality) clustering, which we will consider as the type 2 method.

**Table 1:** Comparisons between three different types of SNV analysis methods: (1) one-by-one SNP selection methods, (2) genotype-demultiplexing methods, and (3) VAE-based cell-centric methods

Type of methods	Examples	Purpose	Statistical tool	Suitability for lineage tracing data
One-by-one SNP selection methods	Monovar [10] SCAN-SNV [11] Conbase [12] Monopogen [13] mgatk [14] MQuad [15]	Identify a few SNPs that are non-randomly distributed and likely to be biologically functional or informative	Ranking of *p*-value or other probability metric	Not suitable because the same SNP can appear inmultiple close lineages in lineage tracing data
Genotype-demultiplexing methods	SCG [16] BnpC [17] Cardelino [18] VireoSNP [19] Souporcell [20]	Demultiplex single cells from different genotypes in somatic mutation or donor mixing experiments	Mixture model involvingvectors of SNPs	Not suitable because optimal vectors of SNPs in lineage tracing data are highly correlated soconvergence problems easily occur
VAE-based cell-centric methods	SNPmanifold^a^ bmVAE [21]	Cluster single cells to neighboring cells with similar genotypes in general SNV mutation data	Learning a better cell-cell distance metric using VAE	Suitable because VAE does not suffer convergence problems and performs well even in high-covariance lineage tracing data

^a^SNPmanifold is better than bmVAE (dedicated for binary genotypes) [21] in terms of lineage accuracy in scRNA-seq data with higher read depths and continuous allele frequency (Supp. Figs. 1, 8)

The first type of statistical method (one-by-one SNP selection methods) is designed to identify a few SNPs that are non-randomly distributed and likely to be biologically functional or informative. This type of method often devises a new *p*-value (or other probability metrics) and rank informative SNPs based on this *p*-value (or other probability metrics). Nuclear SNP selection methods include Monovar [10] which detects variants with a high posterior probability of more than one allele in the cell population, and more recent methods by leveraging phasing with germline variants to reduce technical false positives [11–13]. Mitochondrial SNP selection methods include mgatk by using variance-mean-ratio on raw allele frequency in the cell population [14], and MQuad by using BIC (Bayesian information criterion) to select variants supporting more than one clone [15].

The second type of statistical method (genotype-demultiplexing methods) is designed to demultiplex (i.e., identify the origins of) single cells from different genotypes in somatic mutation or donor-mixing experiments. Statistically, this type of method optimizes vectors of SNPs, each representing 1 distinct genotype, and assigns each single cell to one of the vectors in a mixture model fashion. Example genotype-demultiplexing methods include Bayesian mixture models with Bernoulli noise (e.g., SCG Single-Cell Genotyper and BnpC) [16, 17], Cardelino that uses a putative phylogenetic tree as a prior and supports binomial noise for scRNA-seq data [18], and methods with higher computational efficiency for demultiplexing donors with germline variants [19, 20].

Recently, the natural, random passenger DNA mutations (e.g., SNVs) have been utilized as genetic markers for single-cell lineage tracing [9, 14, 22–25]. The core idea is that if a mutation occurs in an ancestor cell, all of its descendant cells will also carry this same mutation. After several rounds of mutations at different timepoints, a hierarchical structure of mutation will form, and this hierarchy is informative of the evolutionary history of different ancestral lineages. Nevertheless, this type of lineage tracing data has high covariance of different SNPs because different lineages are closely related, so both types of existing SNV analysis methods discussed in the previous paragraphs (one-by-one SNP selection methods and genotype-demultiplexing methods) are not suitable to use (Table 1). One-by-one SNP selection methods (type 1) are not suitable because SNPs in lineage tracing data often appear in more than one lineage, so individual SNPs are not fully representative of the hierarchy of mutation patterns. Genotype-demultiplexing methods (type 2) are not suitable because they involve numerical steps of directly optimizing vectors of SNPs representing cell clusters. They may suffer from convergence problems when vectors of SNPs representing different lineages are highly coherent and correlated in lineage tracing data. This is a generic local-optima challenge for model-based clustering on high-dimensional data, especially with (a large number of) imbalanced clusters in lineage tracing data.

To develop a SNV analysis method that is suitable for high-covariance single-cell lineage tracing data, we were inspired by recent successes of VAE (variational autoencoder) [26], a kind of generative factor analysis neural network model, in solving numerous challenging biological problems, such as predicting cell transition [27–29] and integrating knowledge from different omics [30]. Conventional cell-cell distance metric in genetic analysis is based on binarized genotypes or allele frequency, which are highly sensitive when the coverage is extremely low, like in scRNA-seq data. Here, we tried to adapt VAE to SNV mutation data with higher read depths and continuous allele frequency (e.g., scRNA-seq, mitochondrial DNA). We aimed to use VAE to learn a better (i.e., denoised, efficient, and interpretable) cell-cell distance metric on a lower dimensional space that captures covariance of different SNPs in single-cell lineage tracing data. Then, we performed cell clustering based on this lower-dimensional space that supports a more effective cell-cell distance metric. By doing this, problematic numerical steps involving high covariance of SNPs can be largely avoided, leading to more accurate clustering. Also, visualization of hierarchical mutation patterns (e.g., mutation gaps and protruding lineages) becomes clear under UMAP of embedding manifold based on this better cell-cell distance metric.

**Fig. 1:**
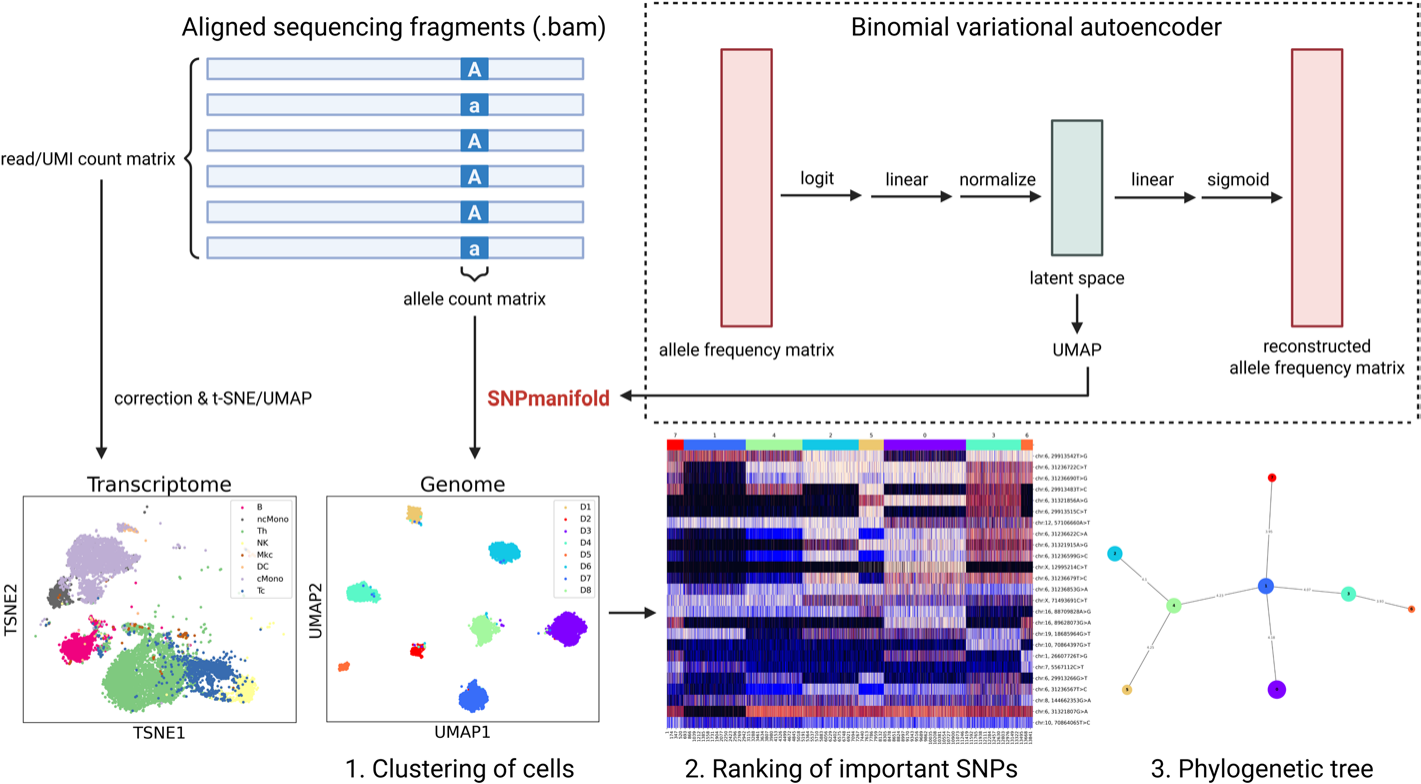
Schematic showing the workflow of SNPmanifold in a donor-multiplexing scRNA-seq dataset (Donor8). After performing single-cell sequencing and obtaining a BAM file, researchers normally analyze the transcriptomic part by compiling a read/UMI count matrix and transforming it to an informative transcriptomic geometrical manifold using t-SNE or UMAP. SNPmanifold offers a genomic analog to this. After compiling an allele count matrix, SNPmanifold transforms it into an informative SNV geometrical manifold using a binomial variational autoencoder and UMAP. SNPmanifold then performs three downstream analyses on the SNV geometrical manifold: (1) Clustering of cells with similar genotypes (in latent space), (2) ranking of important SNPs (via *F*-test), and (3) Constructing the phylogenetic tree (via neighbor-joining method). SNPmanifold can visualize SNV mutation hierarchy in the same way t-SNE or UMAP can visualize cell-type hierarchy for transcriptomics

In this work, we introduce SNPmanifold (Fig. 1), a Python package dedicated to convenient analysis and straightforward interpretation of the latent space in VAE (upper right of Fig. 1). SNPmanifold performs three downstream analyses on the SNV embedding manifold: (1) clustering of cells with similar genotypes (in latent space), (2) ranking of important SNPs (via *F*-test), and (3) constructing the phylogenetic tree (via a neighbor-joining method). By learning a better cell-cell distance metric, these three standard tasks can benefit from effective strategies used in single-cell transcriptomic analysis, including exploration via visual examination and continuous space analysis, such as graph-based clustering. Overall, SNPmanifold is a single-cell SNV analysis tool that is suitable for complex, flexible single-cell SNV mutation data with continuous allele frequency, such as in the context of lineage tracing. In the following sections, we will demonstrate how SNPmanifold can reveal insights into single-cell clonality and lineages more accurately and comprehensively than other existing statistical methods, from simpler scenarios of demultiplexing a large number of donors via germline nuclear SNPs to high-covarince scenarios of revealing somatic lineages via mitochondrial SNVs in blood or cancer cell lines and primary human samples.

## Results

### Overview of SNPmanifold and hyperparameter optimization of variational autoencoder for continuous allele frequency

VAE (variational autoencoder) is a kind of generative factor analysis neural network model that learns the generative process to reconstruct inputs from raw inputs. It typically consists of an encoder, a neural network with multiple progressively narrowing latent layers, and a decoder, a neural network with multiple progressively widening latent layers. Between the encoder and the decoder, there is one single narrowest hidden layer called the latent space, which theoretically captures the most informative features of raw input data. When the prior in the latent space is Gaussian, this model is called a deep latent Gaussian model or DLGM. Demonstrated by previous studies [27–30] on VAE in solving other biological problems, this latent space can effectively identify covariances in the distribution of input data that are unclear in raw space and also correct missing values in individual input data, provided that the structure of VAE and its training strategy are well-optimized. This motivated us to adapt VAE with a standard Gaussian prior to SNV mutation data with higher read depths and continuous allele frequency (e.g., scRNA-seq, mitochondrial DNA), and use it as the backbone statistical model for SNPmanifold. As a type of representation learning, the lower-dimensional (generalized linear) space enables learning a better (denoised, efficient, and interpretable) cell-cell distance metric for single-cell SNV lineage tracing data. Based on this better cell-cell distance metric, SNPmanifold can perform cell clustering accurately even in high-covariance lineage tracing context, where other existing methods easily suffer convergence problems due to high covariance of different SNPs.

To optimize the structure of VAE for single-cell SNV lineage tracing data, we first replaced the Gaussian likelihood cost function in a typical VAE with a binomial likelihood cost function, which better describes the statistical distribution of allele counts in single-cell mutation data, particularly considering the coverage is generally low with a large proportion of zeros. We then systematically varied hyperparameters of binomial VAE to evaluate the performance in resolving ground-truth single-cell SNV lineages. Three hyperparameters are involved: (1) number of hidden layers (LeakyReLU activation in between layers; Supp. Figs. S1, S2), (2) value of $\beta$ (strength of standard Gaussian prior; Supp. Figs. S1, S3), and (3) number of latent dimensions D of embedding manifold (Supp. Figs. S1, S4). We also tried to apply optimized hyperparameters of bmVAE [21] (another type 3 method which focuses on binary genotypes in tumor) to our binomial VAE for continuous allele frequency (Supp. Figs. S1, S2). We tested VAEs with different hyperparameters using Donor4, Donor8, and Donor18 ground-truth datasets (Supp. Fig. S5). These 3 donor-multiplexing scRNA-seq datasets consist of single cells and nuclear germline SNPs from 4, 8, and 18 human donors respectively. We benchmarked these results (Supp. Fig. S1) quantitatively using 2 metrics: 1. Accuracy of clustering to ground-truth donor labels, and 2. Silhouette score of ground-truth donor labels (a metric for compactness of clusters, a higher score indicates better results; difference between average cell-pair distance within the same donor and average cell-pair distance to another nearest donor). *K*-means clustering is used for Donor4 and Donor8 datasets, while Leiden clustering (graph-based) is used for Donor18 dataset.

According to benchmarking results (Supp. Fig. S1), for the number of hidden layers, VAE with 1 hidden layer outperforms deeper VAEs (3 hidden layers, 5 hidden layers, bmVAE hyperparameters) significantly in terms of accuracy and silhouette score, especially on Donor4 dataset (possibly due to lower number of cells and overfitting can be significant with deeper models). For the value of $\beta$, VAEs with different values of $\beta$ = (0, 0.5, 1, 1.5, 2) show similar accuracies and silhouette scores. For the number of latent dimensions D of the embedding manifold, VAEs with higher values of D = (no. of SNPs/(2, 10)) show better accuracies and silhouette scores than VAEs with lower values of D = (no. of SNPs/(30, 50, 100)).

On top of this, we further proposed an “observed-SNP normalization” between the encoder and the latent space (upper-right of Fig. 1). This normalization is different from batch normalization and layer normalization. It divides the latent factor of each cell by the number of observed SNPs in that cell (i.e., cell-specific division by the number of observed SNPs: sum(DP > 0)). We believe that this can help correct the effect of imbalanced coverage between cells with the same genotype (Supp. Fig. S6). According to benchmarking results (Supp. Fig. S1), VAE with “observed-SNP normalization” shows better accuracy and silhouette score in Donor8 dataset, a dataset with higher cell type diversity (peripheral blood mononuclear cells).

We therefore suggested binomial VAE with 1 hidden layer, $\beta$ = 0, D = no. of SNPs/2, and “observed-SNP normalization” to be the final backbone model for SNPmanifold. Of note, our model shows robust performance with varying $\beta$ and we kept the original default $\beta =0$ throughout for simplicity. Considering potential regularization benefits of small $\beta$ in some datasets, we keep $\beta$ to be a tunable argument for users to specify in SNPmanifold. Based on the better cell-cell distance metric learnt by this VAE, SNPmanifold performs three downstream analyses: (1) clustering of cells with similar genotypes (in latent space), (2) ranking of important SNPs (via *F*-test), and (3) constructing the phylogenetic tree (via neighbor-joining method).

In the 3 aforementioned benchmarking donor-multiplexing scRNA-seq datasets, SNPmanifold has significantly better accuracy and silhouette score than UMAP alone and bmVAE [21] package (Supp. Figs. S1, S7, S8). Compared to UMAP alone on the allele frequency space of all SNPs, which assigns uniform weights to different SNPs, SNPmanifold assigns different weights to different SNPs; thus, it can highlight lineage-specific SNPs for better clustering. Compared to bmVAE package which binarizes genotypes, SNPmanifold performs analyses based on continuous allele frequency; therefore, it is more suitable for mutation data with higher read depths (e.g., scRNA-seq, mitochondrial DNA).

### SNPmanifold projects multiplexed donors of origin to multiple disconnected manifolds based on nuclear SNPs

After finalizing the structure of VAE in SNPmanifold, we first applied SNPmanifold to 3 donor-muliplexing datasets using germline nuclear SNPs (Donor4, Donor8, Donor18; Supp. Fig. S5). These 3 datasets mimic mixes of distant cell lineages and have ground truths (identity of donors), so they are suitable for initial demonstration of the effectiveness of SNPmanifold in identifying and visualizing SNV lineages.

**Fig. 2:**
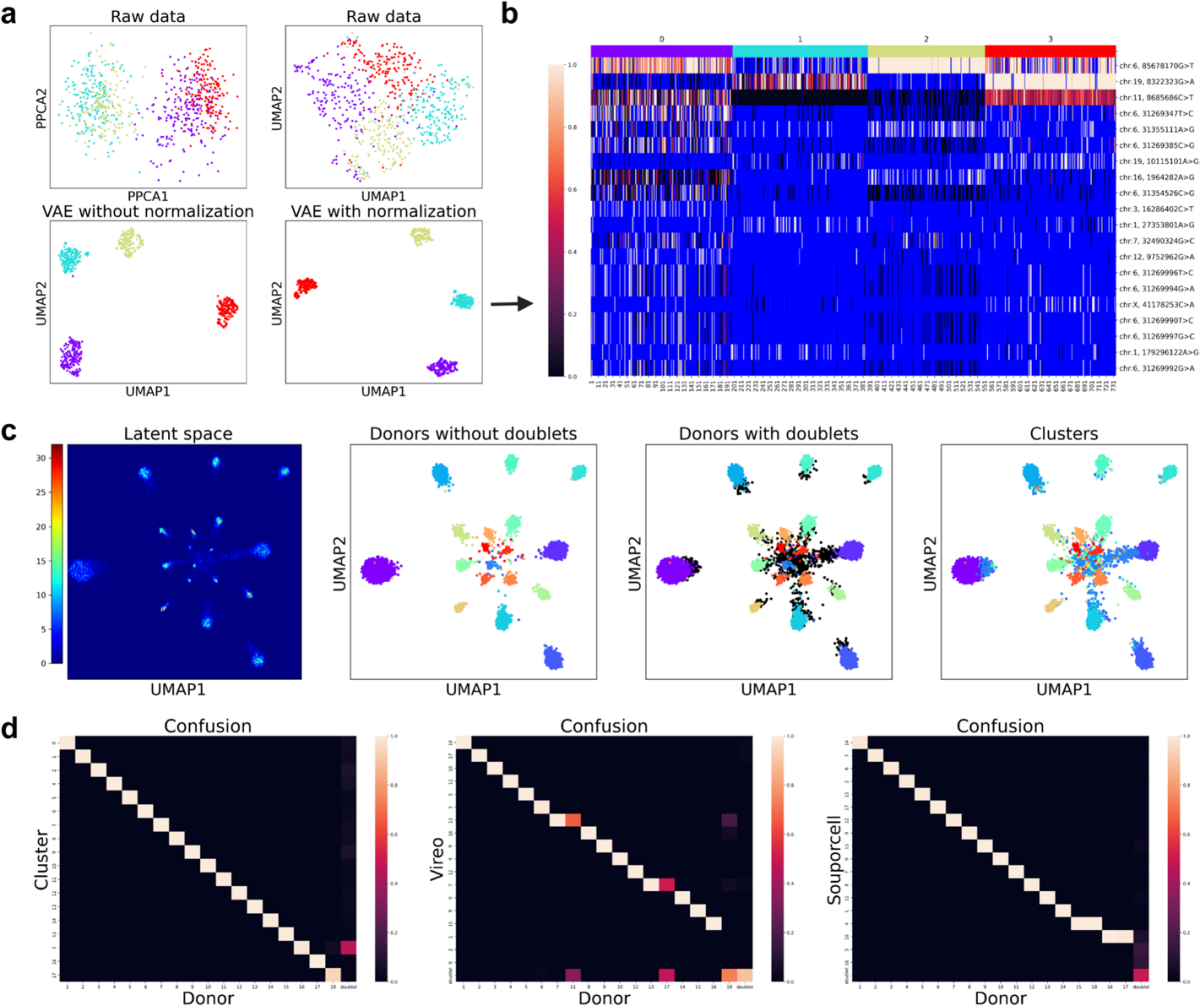
SNPmanifold demultiplexes scRNA-seq with a large number of donors accurately and visualizes them in disconnected manifolds. **a** In scatter plots colored by ground-truth donor labels, SNPmanifold (VAE with “observed-SNP normalization”) resolves single cells in Donor4 dataset into 4 disconnected manifolds better compared to PPCA, UMAP of the raw matrix, and VAE without “observed-SNP normalization.” **b** Heatmap of allele frequency matrix shows 4 distinct genotypes for 4 SNPmanifold clusters (by *k*-means clustering). The blue color indicates missing values (i.e., DP = 0). **c** In density plot and scatter plots colored by ground-truth donor labels and SNPmanifold cluster labels (by graph-based Leiden clustering), SNPmanifold resolves single cells in Donor18 dataset into 18 disconnected manifolds. **d** Confusion matrices between donor labels and cluster labels (from left to right: SNPmanifold, Vireo, Souporcell) of Donor18 dataset. The values of each column sum to 1. In the absence of genotype reference, the accuracy of SNPmanifold clusters (left) reaches 99.3$\%$ and outperforms Vireo (middle) and Souporcell (right), two published type 2 donor-demultiplexing statistical methods

We first applied SNPmanifold to Vireo’s tutorial dataset (Donor4) [19], a scRNA-seq dataset which multiplexes 734 single cells with 1905 germline nuclear SNPs from 4 donors (Fig. 2a, b, Supp. Fig. S9). SNPmanifold (VAE with “observed-SNP normalization”) resolves single cells into 4 disconnected manifolds better compared to PPCA, UMAP of the raw AF matrix, and VAE without “observed-SNP normalization” (Fig. 2a). *K*-means clustering of the latent space (Supp. Fig. S9a) identifies 4 donors successfully with good clustering metrics (distortion and silhouette score) and an accuracy of 99.8$\%$ compared to ground-truth donor labels (Supp. Fig. S9b; assigned by Vireo [19] using reference to genotypes, a published type 2 donor-demultiplexing statistical method).

Next, we applied SNPmanifold to Demuxlet’s benchmarking dataset (Donor8) [31], a scRNA-seq dataset which multiplexes 13,939 PBMCs (peripheral blood mononuclear cells) with 929 germline nuclear SNPs from 8 donors (Fig. 1, Supp. Fig. S10). VAE resolves single cells into 8 disconnected manifolds (Fig. 1), and *k*-means clustering of the latent space (Fig. 1, Supp. Fig. S10a) identifies 8 donors successfully with good clustering metrics (distortion and silhouette score) and an accuracy of 99.7$\%$ compared to ground-truth donor labels (Supp. Fig. S10b; assigned by Demuxlet [31] with reference to genotypes in its original report as a type 2 donor-demultiplexing statistical method).

Lastly, we applied SNPmanifold to a challenging dataset (Donor18) [32], a scRNA-seq dataset which multiplexes 9436 iPSCs (induced pluripotent stem cells) with 864 germline nuclear SNPs from 18 donors (Fig. 2c, d, Supp. Figs. S11, S12). Despite severely imbalanced sample sizes across donors (67 to 1519 cells; Supp. Fig. S11c), VAE resolves single cells into 18 disconnected manifolds (Fig. 2c), and Leiden clustering (graph-based) of the latent space (Fig. 2c) identifies 18 donors successfully with an accuracy of 99.3$\%$ compared to ground-truth donor labels (Fig. 2d; generated by using known reference genotypes via Vireo [19]). In the absence of genotype reference, the accuracy of SNPmanifold outperforms two published type 2 donor-demultiplexing statistical methods (Vireo [19] and Souporcell [20]; Fig. 2d, Supp. Fig. S12), particularly for donors with small sample sizes. This dataset highlights the strength of SNPmanifold for being extra sensitive and accurate to lineages with small numbers of cells, even in a dataset with a large number of lineages.

### SNPmanifold projects lineages of somatic mitochondrial clones to multiple disconnected tree-like manifolds

After initial successes of SNPmanifold in 3 donor-multiplexing datasets, we would like to demonstrate the capability of SNPmanifold in identifying hierarchical lineages of somatic SNV clones within individual cell lines using mitochondrial SNPs (TF1$\_$GM11906, MKN45; Supp. Fig. S5). We focused on mitochondrial SNPs because they are currently one of the most popular choices for single-cell lineage tracing in biomedical research [9, 14, 22] and we would like to demonstrate the applicability of SNPmanifold to this popular technology. In terms of advantages, mitochondrial SNPs have fewer positions, higher sequencing depths, and higher mutation rates, making them suitable for somatic lineage tracing via continuous allele frequency without any need for in vivo biochemical manipulation.

**Fig. 3:**
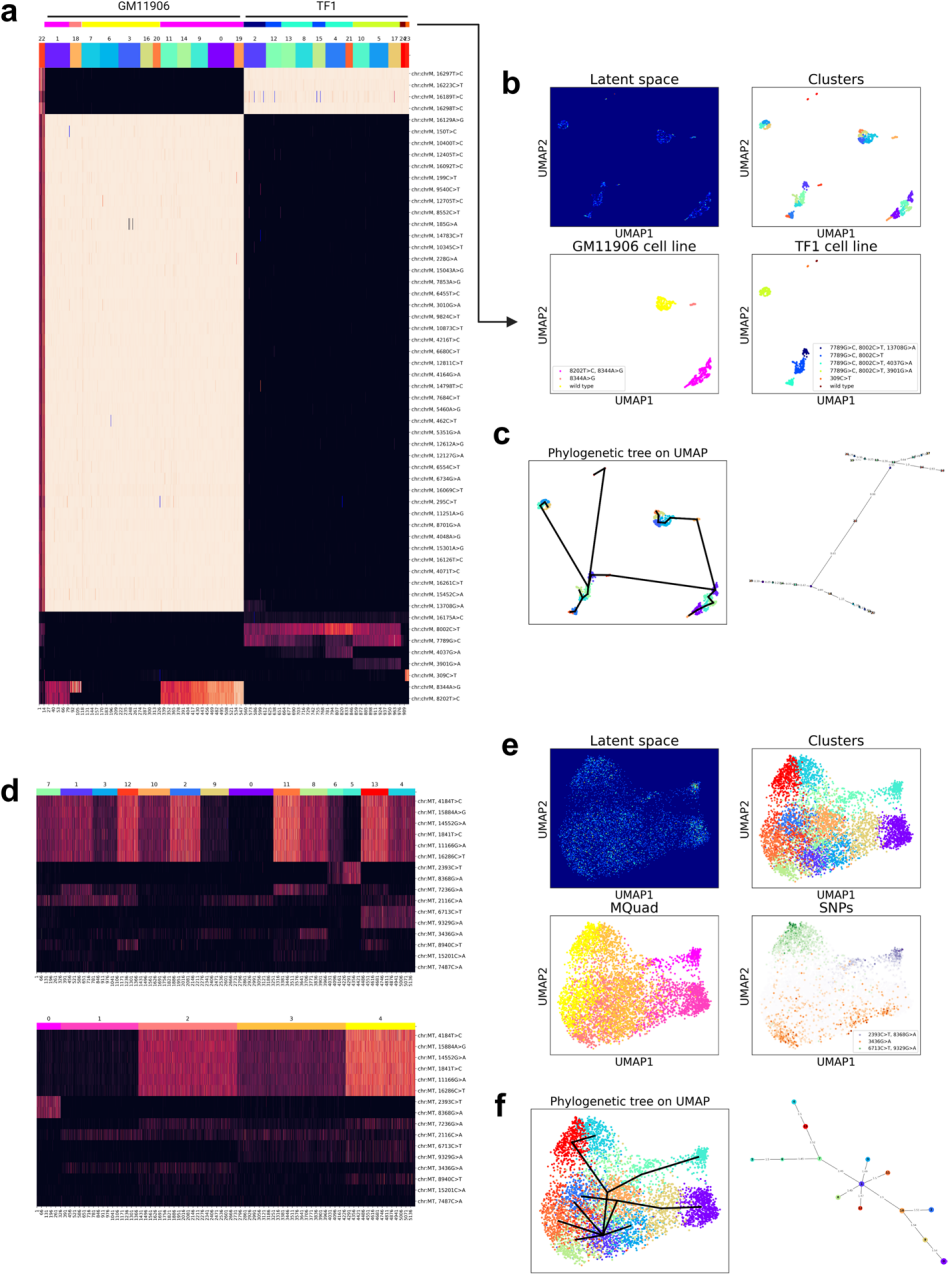
SNPmanifold identifies somatic mitochondrial lineages and their hierarchical phylogeny within cell lines. **a** Heatmap of allele frequency matrix shows how SNPmanifold resolves mitochondrial genotypes in TF1$\_$GM11906 dataset. There are 47 nearly homozygous SNPs shown on top which correspond to germline mutations of the two cell lines, and several cell-line-specific somatic SNPs with continuous allele frequency shown at the bottom. **b** In density plot and scatter plots colored by cluster labels and cell-line labels, SNPmaniold resolves single cells into 8 disconnected manifolds (putative lineages; 4 for TF1 cell line, 3 for GM11906 cell line, 1 for doublets). **c** Phylogenetic tree of TF1$\_$GM11906 dataset. **d** Heatmaps of allele frequency matrix show how SNPmanifold (upper) resolves weak mitochondrial SNPs (shown at the bottom) better than MQuad (lower) in the MKN45 dataset. **e** In density plot and scatter plots colored by different labels, SNPmanifold resolves single cells into 2 disconnected manifolds: upper-right and lower-left. The upper-right manifold is distinctly separated in mitochondrial allele frequency and correlated with copy number loss of chr4q. The lower-left manifold represents a tree-like complex lineage with fluctuating allele frequency of multiple mitochondrial SNPs. **f** Phylogenetic tree of MKN45 dataset. Utilizing continuous allele frequency, SNPmanifold can highlight more putative phylogenetic lineages than more discretized methods

We first applied SNPmanifold to mtscATAC-seq’s benchmarking dataset (TF1$\_$GM11906) [14], a single-cell mitochondrial ATAC-seq dataset which mixes 1001 single cells with 56 mitochondrial SNPs from two hematopoietic cell lines (TF1 and GM11906 cell lines; Fig. 3a–c, Supp. Figs. S13, S14). VAE resolves single cells into 8 disconnected manifolds (putative lineages; Fig. 3b): 4 correspond to TF1 cell line (Fig. 3a, bottom right of Fig. 3b), 3 correspond to GM11906 cell line (Fig. 3a, bottom left of Fig. 3b), and 1 corresponds to doublets of the two cell lines (cluster 22; Fig. 3a). According to heatmap of allele frequency matrix (Fig. 3a), this clustering result is based on a set of 47 nearly homozygous SNPs shown on top which separate the two cell lines, and several cell-line-specific somatic SNPs with continuous allele frequency shown at the bottom. All lineages identified by SNPmanifold show good concordance to the top somatic SNPs reported by the original paper using mgatk [14] (a published type 1 method for selecting mitochondrial SNPs). In terms of phylogeny, SNPmanifold constructs a cluster phylogenetic tree (Fig. 3c) in the latent space. This tree shows a reasonable phylogeny where TF1 cell line clusters and GM11906 cell line clusters are separated into two sides of the tree, connected by the cluster of doublets in the middle. Altogether, this dataset simulates a case where two distant genetic lineages undergo somatic mutations individually, and SNPmanifold is capable of summarizing major germline mutations and minor somatic mutations together into one reasonable hierarchical mutation pattern.

Next, we applied SNPmanifold to MQuad’s benchmarking dataset (MKN45) [15, 33], a scDNA-seq dataset which contains 5199 single cells with 322 SNPs from MKN45 human gastric cancer cell line (Fig. 3d–f, Supp. Figs. S15, S16). VAE resolves single cells into 2 disconnected manifolds, 1 upper-right small manifold and 1 lower-left large tree-like manifold (Fig. 3e). The upper-right small manifold represents a simple lineage with mutation 2393C>T&8368G>A (Fig. 3d, e). This mitochondrial lineage shows a good correlation with copy number loss of chr4q (67.1$\%$ of -chr4q cells belong to SNPmanifold clusters 5 and 6, odds ratio: 36.8). On the other hand, the lower-left large manifold represents a tree-like complex lineage (Fig. 3e). According to heatmap of allele frequency matrix (Fig. 3d), this tree-like lineage possesses a varying major haplotype of 6 SNPs shown on top and occasional somatic SNPs shown at the bottom. Compared to MQuad [15] (a published type 1 method for selecting mitochondrial SNPs; followed by Vireo [19]), SNPmanifold shows better resolution to mitochondrial SNPs with weak allele frequency (e.g.. 7236G>A, 2116C>A, etc.; Fig. 3d, e, Supp. Fig. S15a, c). This extra resolution allows SNPmanifold to highlight more putative phylogenetic lineages based on the same mitochondrial mutation profile (Fig. 3f). In short, this dataset demonstrates how SNPmanifold can utilize continuous allele frequency of multiple mitochondrial SNPs to highlight a distinctly separated biological clone and infer putative phylogenetic lineages that can be missed by more discretized methods.

### SNPmanifold reveals correlations between cellular phenotypes and phylogeny of somatic mitochondrial genotypes

Encouraged by the successes of SNPmanifold in identifying lineages of somatic SNV clones within individual cell lines, we would like to further demonstrate its capability in identifying lineages of somatic SNV clones within primary human samples using mitochondrial SNPs (BPDCN, HSPC$\_$PBMC; Supp. Fig. S5). In particular, we would like to show that mitochondrial lineages identified by SNPmanifold are biased to certain cell types because primary human samples have greater cell-type diversity compared to cell lines.

**Fig. 4:**
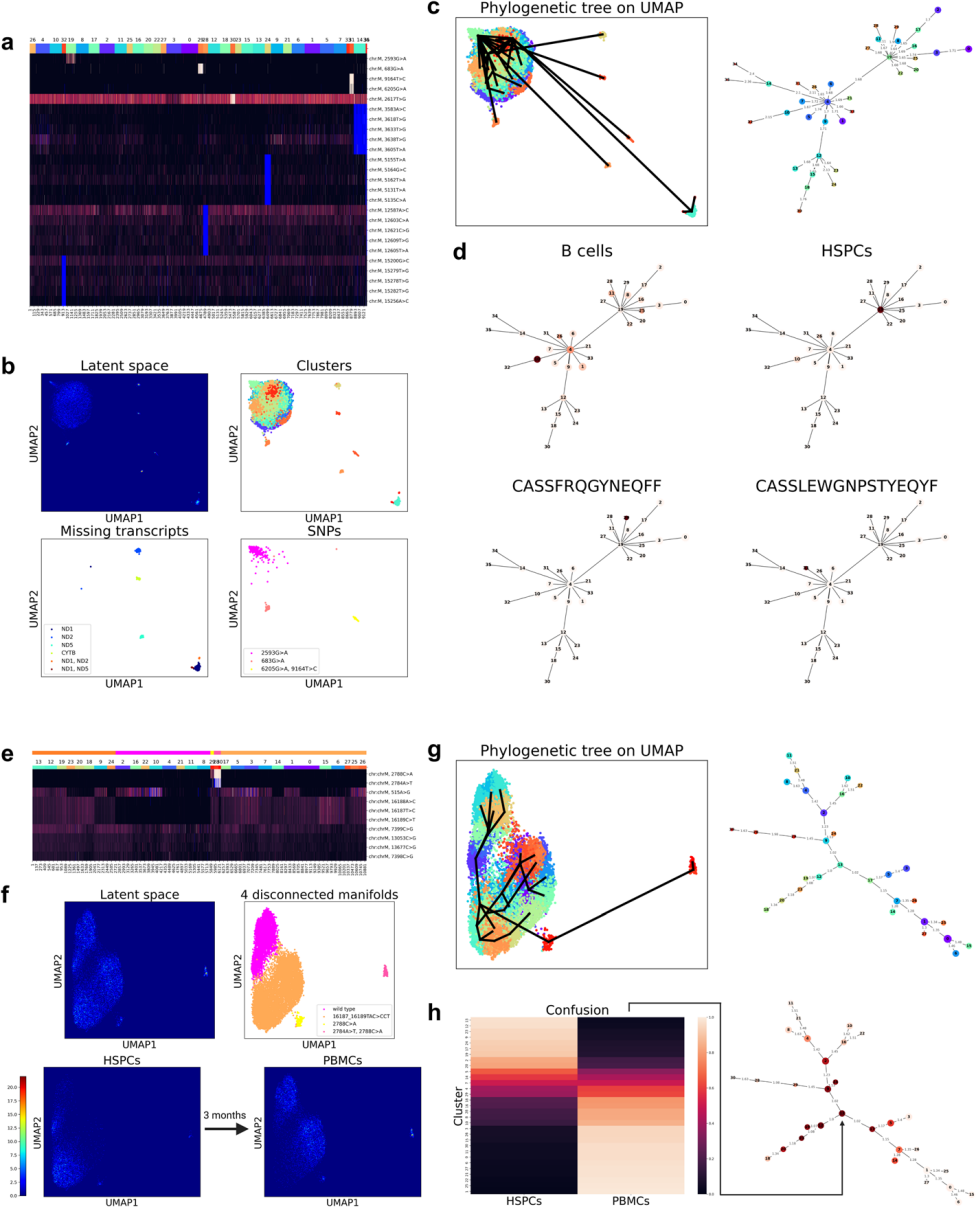
SNPmanifold identifies biologically relevant somatic mitochondrial lineages in primary human samples. **a** Heatmap of allele frequency matrix shows how SNPmanifold resolves mitochondrial genotypes in BPDCN dataset. There are clusters corresponding to strong mutations shown on top and missing mitochondrial transcripts shown in blue color (i.e., DP = 0). **b** In density plot and scatter plots colored by different labels, clusters with strong mutations and missing mitochondrial transcripts are visualized as disconnected manifolds. **c** Phylogenetic tree of BPDCN dataset. **d** By coloring relative abundance on the phylogenetic tree (deeper color means higher abundance), the tree shows bias of cell types (upper) and TRB clonotypes (lower) to certain clusters. More cell types and TRB clonotypes are shown in Supp. Fig. S18. **e** Heatmap of allele frequency matrix shows how SNPmanifold resolves mitochondrial genotypes in HSPC$\_$PBMC dataset based on strong mutations and weak mutations. Blue color indicates missing values (i.e., DP = 0). **f** In density plot and scatter plots, HSPCs and PBMCs (from the same healthy donor; collected 3 months after HSPCs) are spread across 4 disconnected manifolds. They locate at different parts on each manifold, indicating a gradual change in mitochondrial genotype from HSPCs to PBMCs. **g** Phylogenetic tree of HSPC$\_$PBMC dataset. **h** By coloring relative abundance on phylogenetic tree (deeper color means higher abundance), the tree shows bias of HSPCs to the middle of the phylogeny and PBMCs to the periphery. This describes a proliferation event where the same group of HSPCs gradually differentiates into different lineages of PBMCs

We first applied SNPmanifold to MAESTER’s benchmarking dataset (blastic plasmacytoid dendritic cell neoplasm; BPDCN) [9], a single-cell mitochondrial RNA-seq dataset that contains 9204 PBMCs (peripheral blood mononuclear cells) with 274 SNPs from a patient with clonal hematopoiesis (Fig. 4a–d, Supp. Figs. S17–S19). VAE resolves single cells into many disconnected manifolds (putative lineages; Fig. 4b): 6 of them represent missing mitochondrial transcripts and 3 of them represent strong somatic mutations 2593G>A, 683G>A, 6205G>A&9164T>C (Fig. 4a, b, Supp. Fig. S17a). Notably, all 3 mitochondrial lineages with strong somatic mutations (i.e., distinctly separated mitochondrial allele frequency) are biologically relevant (Fig. 4d, Supp. S17c, d, S18): cluster 19 (with 2593G>A) is enriched with HSPCs (hematopoietic stem and progenitor cells) and myeloid cell types (odds ratio: 15.7); 93.8$\%$ of cells in cluster 29 (with 683G>A) are T cells with TRB-clonotype CASSFRQGYNEQFF; 92.4$\%$ of cells in cluster 31 (with 6205G>A&9164T>C) are T cells with TRB-clonotype CASSLEWGNPSTYEQYF. In terms of phylogeny, SNPmanifold constructs a cluster phylogenetic tree (Fig. 4c) in the latent space and identifies 3 major common ancestors (clusters 4, 12, 19), each of which is connected to at least 5 other clusters. The existence of these major common ancestors can suggest putative proliferation events where the same group of progenitor cells keeps on differentiating into diverging lineages of descendant cells (e.g., HSPCs for cluster 19, lymphoid cell types for clusters 4 and 12; Fig. 4d, Supp. S17c, d, S18). Altogether, this dataset showcases how SNPmanifold can resolve mitochondrial genotypes within a primary human sample and identify biologically relevant clones and putative proliferation events.

Lastly, we applied SNPmanifold to mtscATAC-seq’s another benchmarking dataset (HSPC$\_$PBMC) [14], a single-cell mitochondrial ATAC-seq dataset which contains 4,339 HSPCs (hematopoietic stem and progenitor cells) and 6,597 PBMCs (peripheral blood mononuclear cells) with 100 SNPs from a healthy donor (Fig. 4e–h, Supp. Figs. S20, S21). PBMCs from the same healthy donor were collected 3 months after HSPCs, so there is sufficient time for HSPCs to differentiate into PBMCs. VAE resolves single cells into 4 disconnected manifolds (Fig. 4f) based on weak mutation 16187$\_$16189TAC>CCT and strong mutations 2784A>T, 2788C>A (Fig. 4e, f, Supp. Fig. S20a). Notably, although HSPCs and PBMCs are spread across all 4 manifolds, they locate at different parts on each manifold (Fig. 4f). This indicates a gradual change in mitochondrial genotype (Fig. 4e, Supp. Fig. S20a) from HSPCs to PBMCs. According to the cluster phylogenetic tree (Fig. 4g, h), HSPCs localize in the middle of the phylogeny while PBMCs localize on the periphery. This describes a proliferation event where the same group of HSPCs gradually differentiates into different lineages of PBMCs (excluding clusters 28, 29, 30 which possess strong mutations). This dataset provides evidence that mitochondrial lineages based on weak mutations can be biologically relevant and highlights the advantage of performing analysis using continuous allele frequency over binarized genotypes.

## Discussion

The primary motivation of our work was to develop a flexible statistical model that can analyze high-covariance single-cell SNV lineage tracing data with higher read depths and continuous allele frequency (e.g., scRNA-seq, mitochondrial DNA). This type of lineage tracing data is becoming increasingly popular in biomedical research of developmental biology [9, 14, 22] and tumor evolution [23–25], yet existing SNV analysis methods fail for this type of data because they were originally developed for simpler experimental settings where covariance of different SNPs is low in different clusters (Table 1). To escape numerical issues brought by high covariance, we proposed to use VAE (variational autoencoder) to learn a better (denoised, efficient, and interpretable) cell-cell distance metric for mutation data empowered by the generalized-linear, lower-dimensional representations. We then performed clustering based on this learnt cell-cell distance metric. Since this cell-cell distance metric already captures covariance of different SNPs, the subsequent clustering algorithm can converge to the global optimum with higher chances compared to high-covariance and sparse raw data. Also, with this lower-dimension-based cell-cell distance metric, SNPmanifold can identify rare or weak SNVs that have high covariance but are traditionally overlooked by other existing methods due to the small number of cells or weak allele frequency.

During the course of development of SNPmanifold, another paper (bmVAE [21]) proposed to use VAE for clustering binary genotypes in tumor scDNA-seq. In terms of model architecture, bmVAE package utilizes a Bernoulli VAE with 5 hidden layers (LeakyReLU in between), $\beta$ = 0.0001, and D = 3. While bmVAE package achieves high accuracy in clustering binary genotypes in tumor mutation data, its VAE model and downstream analyses are not compatible with mutation data with higher read depths and continuous allele frequency (e.g., scRNA-seq, mitochondrial DNA). In the 3 benchmarking donor-multiplexing scRNA-seq datasets (Donor4, Donor8, Donor18), bmVAE package (with binarized matrices) shows worse accuracy and significantly worse silhouette score (a metric for compactness of clusters, a higher score indicates better results) compared to SNPmanifold (Supp. Figs. S1, S8). Here are two possible reasons. Firstly, SNPmanifold performs modeling based on continuous allele frequency, so it can achieve better accuracy by leveraging information that is lost when matrices are binarized (e.g., heterozygous SNPs). Secondly, the VAE of SNPmanifold is generalized linear (except for logit in the beginning and sigmoid in the end, no LeakyReLU; Fig. 1), in contrast to bmVAE which is non-linear with LeakyReLU. Since real biological lineages are usually linearly represented by allele frequency, linear VAE of SNPmanifold is sufficient to describe this type of data with high accuracy, while non-linear VAE (e.g., bmVAE) may overfit non-linear components of scRNA-seq, such as technical artifacts during sequencing and cell-type differences within lineage. In short, through extended modeling of continuous allele frequency, SNPmanifold outperforms bmVAE [21] in donor-multiplexing scRNA-seq data, and is more suitable for mitochondrial lineage tracing data.

Based on the better cell-cell distance metric in VAE, SNPmanifold can visualize SNV mutation hierarchy using UMAP in the same way that UMAP visualizes cell-type hierarchy in single-cell transcriptomics. This is an advantage that other existing numerical SNV-analysis methods do not have, and it can be useful in the context of lineage tracing because important biological features such as the number of distinct somatic lineages and their hierarchical differentiation history can now be straightforwardly examined visually. Before our SNPmanifold, biomedical researchers usually used bifurcating phylogenetic trees (Supp. Figs. S14, S16, S19, S21) to do such analysis, but bifurcating phylogenetic trees cannot visualize multi-branching phylogeny (i.e., one group of progenitor cells differentiates into two or more diverging lineages) that occasionally occur in somatic lineage tracing data (MKN45, BPDCN, HSPC$\_$PBMC; Figs. 3f and 4c, g). Our SNPmanifold provides an alternative multifurcating cluster phylogenetic tree for visualization, so that users can explore bifurcating and multifurcating trees together to understand phylogeny more comprehensively.

At last, we would like to outline some potential usage of our Python package, SNPmanifold, in future biomedical research. SNPmanifold is a VAE-based cell-centric SNV analysis method (Table 1) that is capable of clustering, visualizing, and inferring cluster phylogenetic trees of single-cell SNV lineage tracing data. After performing single-cell lineage tracing experiments, the first step is to choose candidate SNVs. In this work, we chose population-scale common germline nuclear SNPs for identifying lineages from different donors, because they are known to have lineage tracing power in differentiating different individuals [34]. We also chose mitochondrial SNPs for identifying somatic lineages within one individual or one cell line, because they were demonstrated to have a high enough mutation rate and drifting rate to trace healthy somatic lineages that rarely undergo nuclear DNA mutation [9, 14, 22]. It is also one of the more popular choices of lineage tracing sequencing technology. Beyond data types demonstrated in this work, users can choose other relevant candidate SNVs, such as CRISPR-edited sites for CRISPR data [23] and somatic nuclear SNPs for frequently mutated tumor data [35]. For somatic nuclear SNPs, since they usually have low read depths and high dropout rates, we strongly recommend users to first select confident candidate SNPs using state-of-the-art type 1 method (e.g., Monopogen [13]) before detecting lineages using SNPmanifold. Then, users can compile allele count matrices (AD matrix, DP matrix; e.g., using cellsnp-lite [34]) and input them to SNPmanifold. SNPmanifold will then do a pre-filtering of SNPs to retain only informative SNPs with high variance and coverage, and train a VAE to output the latent space. Based on this latent space with better cell-cell distance metric, SNPmanifold will perform clustering, rank important SNPs, construct cluster phylogenetic tree, and visualize SNV mutation hierarchy using UMAP. Users can present these analysis outputs directly in their work after brief modifications of labels and legends.

## Conclusions

In this work, we introduce SNPmanifold, a Python package that learns an SNV embedding manifold using a binomial variational autoencoder to give an efficient and interpretable cell-cell distance metric. We demonstrate that SNPmanifold is a suitable tool for analysis of complex, single-cell SNV mutation data, such as in the context of demultiplexing a large number of donors and somatic lineage tracing via mitochondrial SNV data and can reveal insights into single-cell clonality and lineages more accurately and comprehensively than existing methods. We envision that SNPmanifold will greatly facilitate comprehensive studies of single-cell SNV lineages (e.g., scRNA-seq, mitochondrial sequencing), thereby advancing our understanding of dynamical biological systems such as hematopoiesis and tumor evolution at single-cell resolution.

## Methods

### Binomial variational autoencoder

VAE (variational autoencoder) is a kind of generative factor analysis neural network model that learns the generative process to reconstruct inputs from raw inputs. When the prior in the latent space is Gaussian, this model is called a deep latent Gaussian model or DLGM. SNPmanifold uses a shallow binomial VAE (variational autoencoder) with standard Gaussian prior as the backbone statistical model to learn a better cell-cell distance metric for sparse single-cell SNV lineage tracing data. It takes a cell by SNP allele frequency matrix (AF matrix) $X = A / D$, where its row $\varvec{x}_i$ denotes the allele frequency vector for all selected variants in cell *i*, and A (or AD) and D (or DP) are matrices for counts of the alternative allele and the total count of all alleles, respectively.

The structure of our VAE (upper right of Fig. 1) consists of an encoder and a decoder. The encoder contains a logit transformation (with a smoothing pseudo-count) followed by linear transformation (with parameters $\varvec{\phi }$) and observed-SNP normalization (cell-specific division by the number of observed SNPs: $l_i = \text {sum}(\varvec{d}_i>0$)). The decoder reversely contains a linear transformation (with parameters $\varvec{\theta }$) followed by a sigmoid transformation. After all transformations, it outputs a reconstructed allele frequency matrix $\hat{X}$, as follows (here is for each cell *i*, and we omit the cell index *i* for simplicity):

1 $$\begin{aligned} (\varvec{{\mu _{z}}} , \log (\varvec{\sigma _{z}}^2)) = \text {encoder}_{\varvec{\phi }}(\varvec{x}), \; \hat{\varvec{x}} = \text {decoder}_{\varvec{\theta }}(\varvec{\mu _{z}}). \end{aligned}$$

With the above encoder and decoder setting, we can view this VAE model as a latent model of $\varvec{z}$, for which we can have the likelihood of each cell *i* in the form of binomial distribution by using $\hat{\varvec{x}}_i = \text {decoder}_{\varvec{\theta }}(\varvec{z}_i)$, as follows,

2 $$\begin{aligned} p(\varvec{a}_i | \varvec{d}_i, \varvec{z}_i, \varvec{\theta }) = \prod \limits _{j \in \text {SNP}}\text {Binomial}(a_{i,j} | d_{i,j}, \hat{x}_{i,j}) \propto \prod \limits _{j \in \text {SNP}} \hat{x}_{i,j}^{a_{i,j}} (1-\hat{x}_{i,j})^{d_{i,j}-a_{i,j}} \end{aligned}$$

We aim to approximate its posterior distribution via a variational distribution in the form of a multivariate Gaussian distribution with a diagonal covariance matrix, via using $\varvec{\mu }_i, \log (\varvec{\sigma }^2_i) = \text {encoder}_{\varvec{\phi }}(\varvec{x}_i)$ as follows,

3 $$\begin{aligned} q(\varvec{z}_i| \varvec{\phi }) = \mathcal {N}(\varvec{z}_i | \varvec{\mu }_i, \text {diag}(\varvec{\sigma }^2_i)); \end{aligned}$$

The cost function to optimize is the weighted sum (weight = $\beta$) of the expected negative log of binomial likelihood (i.e., binary cross entropy loss weighted by DP; evaluated through variational inference) and the KL term between the variational distribution and the prior distribution in a standard Gaussian in ordinary VAE:

4 $$\begin{aligned} \text {cost}(\varvec{\phi },\varvec{\theta }) & = \sum \limits _{i\in \text {cell}} -\mathbb {E}_{\varvec{z}\sim q}\left[ \log (p(\varvec{a}_i | \varvec{d}_i, \varvec{z}_i, \varvec{\theta }))\right] + \beta \times D_{KL}(\mathcal {N}(\varvec{\mu }_i, \text {diag}(\varvec{\sigma }^2_i)) \Vert \mathcal {N}(\varvec{0}, \varvec{I})) \nonumber \\ & \propto \sum \limits _{i\in \text {cell}} -\mathbb {E}_{\varvec{z}\sim q}\left[ \sum \limits _{j\in \text {SNP}} a_{i,j}\log (\hat{x}_{i,j})+(d_{i,j}-a_{i,j})\log (1-\hat{x}_{i,j})\right] \nonumber \\ & \quad + \beta \times \sum \limits _{k\in \text {emb}} - 0.5 \times [\log (\sigma _{i,k}^2) - \sigma _{i,k}^2 - \mu _{i,k}^2 + 1] \end{aligned}$$

We optimized hyperparameters using 3 donor-multiplexing scRNA-seq datasets with nuclear germline SNPs (Donor4, Donor8, Donor18; Supp. Fig. S1–S4) and set $\beta$ = 0 and D = 1/2 of the number of input SNPs by default. We then train VAE for 2000 epochs by default using Adam optimizer (learning rate: 0.0001 and weight decay: 0) and encode the input allele frequency matrix into the latent space using the trained encoder. Dropout and weight decay are not currently considered in the training process. After that, SNPmanifold will perform 3 downstream analyses based on the better cell-cell distance metric in the latent space of VAE: (1) clustering of cells with similar genotypes, (2) ranking of important SNPs, and (3) constructing the phylogenetic tree (Fig. 1). In addition, SNPmanifold will visualize SNV mutation hierarchy using UMAP in the same way UMAP visualizes cell-type hierarchy for transcriptomics.

Although we set $\beta$ = 0 by default and the cost function is almost the same as autoencoder, we still call it variational autoencoder because there is a Monte Carlo training step unique to variational autoencoder even when $\beta$ = 0. Also, according to quantitative benchmarking (Supp. Fig. S1) of Donor18 dataset, small $\beta$ = 0.5 may slightly increase the accuracy. Considering potential regularization benefits of small $\beta$ in some datasets, we keep $\beta$ to be a tunable argument for users to specify in SNPmanifold.

### Pre-filtering of SNPs and cells

Before learning a better cell-cell distance metric using VAE, SNPmanifold first performs three pre-filtering steps on input SNPs and cells: (1) number of observed SNPs for each cell, (2) mean coverage of each SNP, and (3) logit-variance of each SNP. SNPmanifold will provide plots on these three quantities to aid users in choosing cut-off for pre-filtering of high-quality SNPs and cells, based on knee points, elbow points, and overall sequencing qualities (Supp. Figs. S22–S29). The motivation of this module is to reduce the size of data and ensure the analysis result is robust and reliable based on high-quality SNPs and cells.

### Clustering of cells with similar genotypes in the latent space

For clustering of cells with similar genotypes, SNPmanifold supports *k*-means clustering and Leiden clustering (graph-based clustering in SCANPY [36]) in either full-dimensional latent space or 3D UMAP [37]. The default method is Leiden clustering in 3D UMAP with resolution 1. If *k*-means clustering is chosen, SNPmanifold will automatically determine the number of clusters based on maximal silhouette score. Users can change clustering method and tune clustering parameter (cluster$\_$no for *k*-means clustering or resolution for Leiden clustering) until the clustering result visualizes well in 2D UMAP [37]. The motivation of this module is to identify cell clusters based on the better cell-cell distance metric in VAE, for subsequent ranking of important SNPs, construction of cluster phylogenetic tree, and other custom cell cluster analysis specified by users.

### Ranking of important SNPs according to cell clusters

For ranking of important SNPs, SNPmanifold uses the cell clusters inferred from the previous module as input and prioritizes SNPs via an ANOVA-like test for each SNP using raw allele frequency. Specifically, it first computes *F*-tests between each cluster and the bulk (i.e., all cells) for each SNP and then ranks SNPs from the lowest *p*-value to the highest *p*-value (Supp. Figs. S9c, S10c, S11b, S13b, S15b, S17b, S20b). Maximum *F*-statistic is capped at 20 and minimum *p*-value is capped at 10^−16^ for computational reasons. In the heatmap of the allele frequency matrix, SNPs are shown in the order of increasing *p*-value for visualization of important SNPs on top. The motivation of this module is to identify SNPs that are weighted importantly in the better cell-cell distance metric in VAE, for double-confirming the reliability of SNV analysis and exploration of possible biological meanings.

### Constructing the phylogenetic tree in the full-dimensional latent space

SNPmanifold constructs both bifurcating phylogenetic tree and multifucating phylogenetic tree. For bifurcating tree, SNPmanifold uses linkage and dendrogram functions in scipy.cluster.hierarchy. For multifurcating tree, SNPmanifold uses the neighbor-joining method to generate a cluster phylogenetic tree (i.e., lineage graph). Specifically, it first computes by default the shortest 100 pairwise distances between cells from each pair of clusters in the full-dimensional latent space, and then iteratively connects neighboring pairs of clusters with the shortest average pairwise distance. In the end, all clusters will be connected into one acyclic graph which is the cluster phylogenetic tree with maximal parsimony in the full-dimensional latent space. In default visualization, the size of each node represents the number of cells in each cluster, and the number on each edge represents the distance between two clusters in the latent space. The motivation of this module is to suggest a putative phylogeny of SNV lineages for straightforward and comprehensive interpretation by biomedical researchers.

## Supplementary Information

Additional file 1: Supplementary figures S1-S29.

Additional file 2: Supplementary tables S1-S3. They are machine-readable xlsx format of Supp. Figs. S1, S5, S22.

## Data Availability

All datasets used in this study are previously published datasets. Donor4 [19] is downloaded from Vireo’s GitHub repository as SNP-count matrices. Donor8 [31] is downloaded from GEO sample GSM2560248 as FASTQs. Donor18 [32] is downloaded from ENA biosample SAMEA6833385 as FASTQs. TF1_GM11906 [14] is downloaded from GEO sample GSM4238432 as FASTQs. MKN45 [33] is downloaded from 10x Genomics as BAM. BPDCN [9] is downloaded from GEO sample GSM5534706 as SNP-count matrices. HSPC_PBMC [14] is downloaded from GEO sample GSM4472965 and GSM4472967 as FASTQs. For the aforementioned FASTQs, we first aligned them to hg19 or hg38 to BAM and then compiled SNP-count matrices using cellsnp-lite [34]. Processed input matrices for SNPmanifold can be downloaded from https://github.com/StatBiomed/SNPmanifold/tree/main/data. SNPmanifold is a standard and open-source Python package with source code freely available at https://github.com/StatBiomed/SNPmanifold [38] and https://doi.org/10.5281/zenodo.17086445 [39] with Apache-2.0 license. For reproducibility, the analysis notebooks and figures are also available in the repository.
