# Supplementary material for: SNPmanifold: detecting single-cell clonality and lineages from single-nucleotide variants using binomial variational autoencoder
Source: Genome Biol. 2025 Sep 26;26:309. doi: 10.1186/s13059-025-03803-3 (PMC12465888; doi:10.1186/s13059-025-03803-3)
Supplement: Supplementary file 1 — Additional file 1: Supplementary figures S1-S29. [file 13059_2025_3803_MOESM1_ESM.docx]

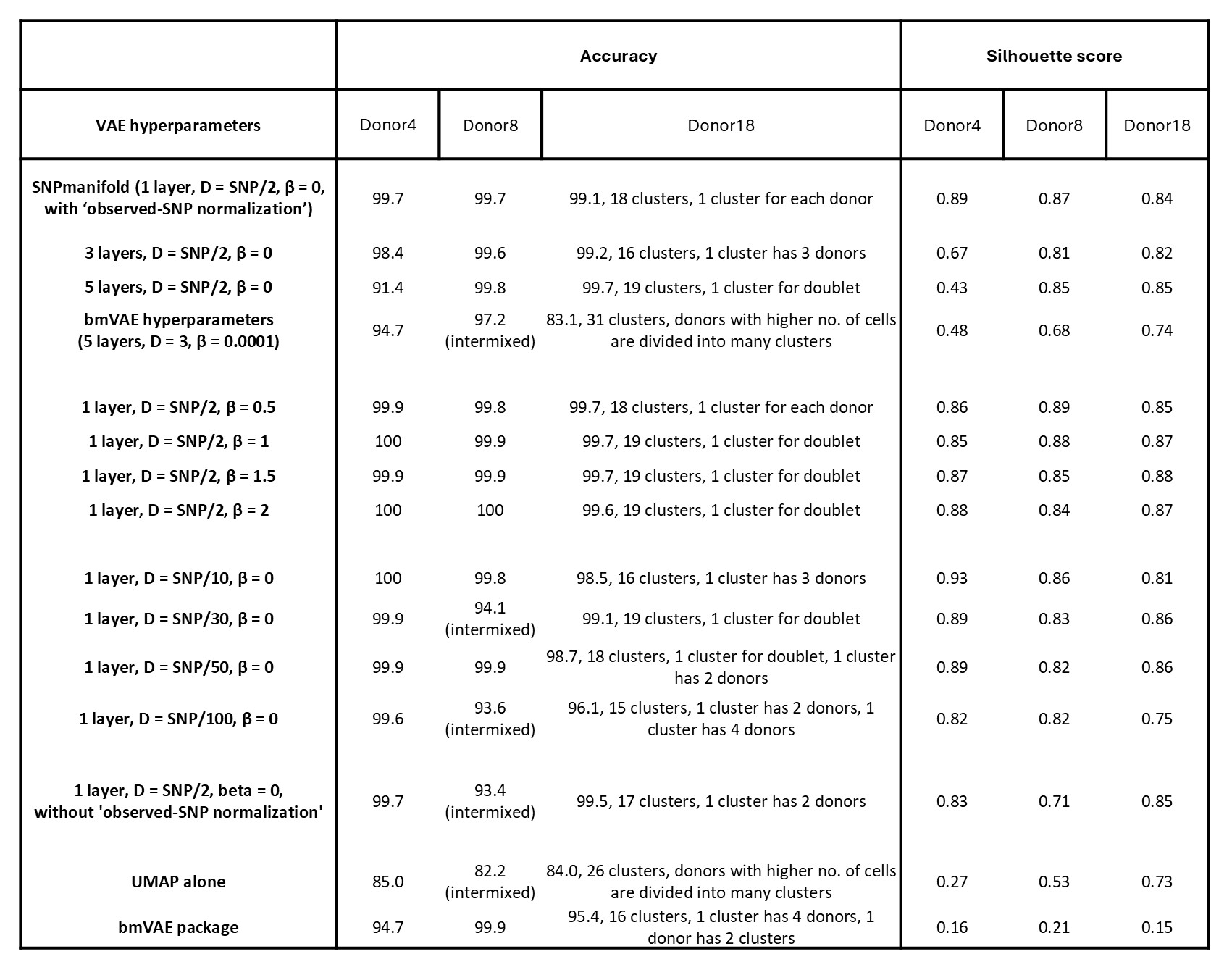


**Supp. Fig. S1.** Benchmarking results (accuracy and silhouette score; higher metrics indicate a better model) of VAEs with different numbers of hidden layers, values of β, numbers of latent dimensions D of embedding manifold, and with or without ‘observed-SNP normalization’, against UMAP alone and bmVAE package. We finally suggested binomial VAE with 1 hidden layer, β = 0, D = no. of SNPs / 2, and ‘observed-SNP normalization’ to be the final backbone model for SNPmanifold. SNPmanifold outperforms UMAP alone and bmVAE package significantly in terms of accuracy and silhouette score in scRNA-seq data with higher read depths and continuous allele frequency. Machine-readable xlsx format can be found in Supp. Table S1.


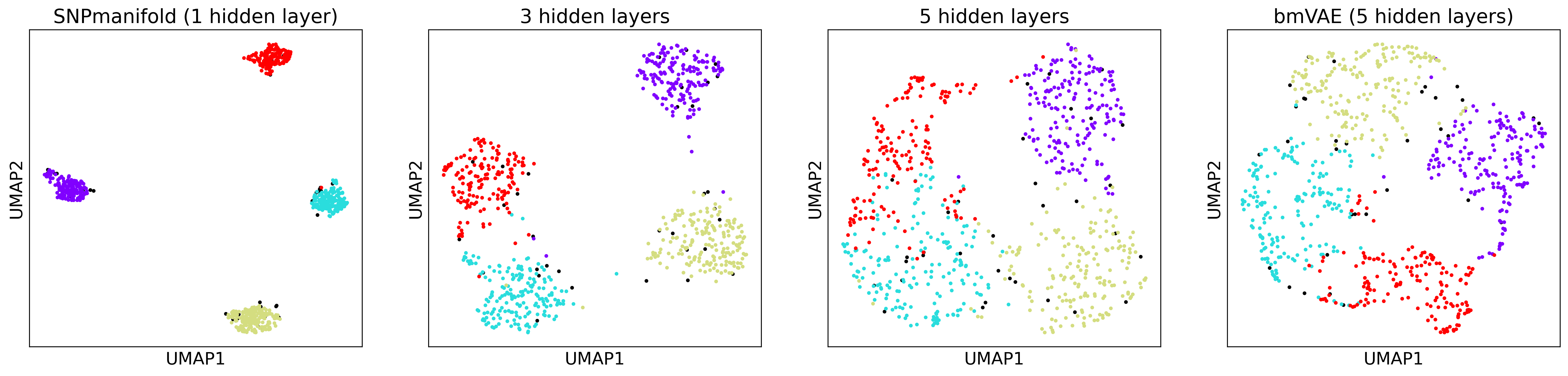

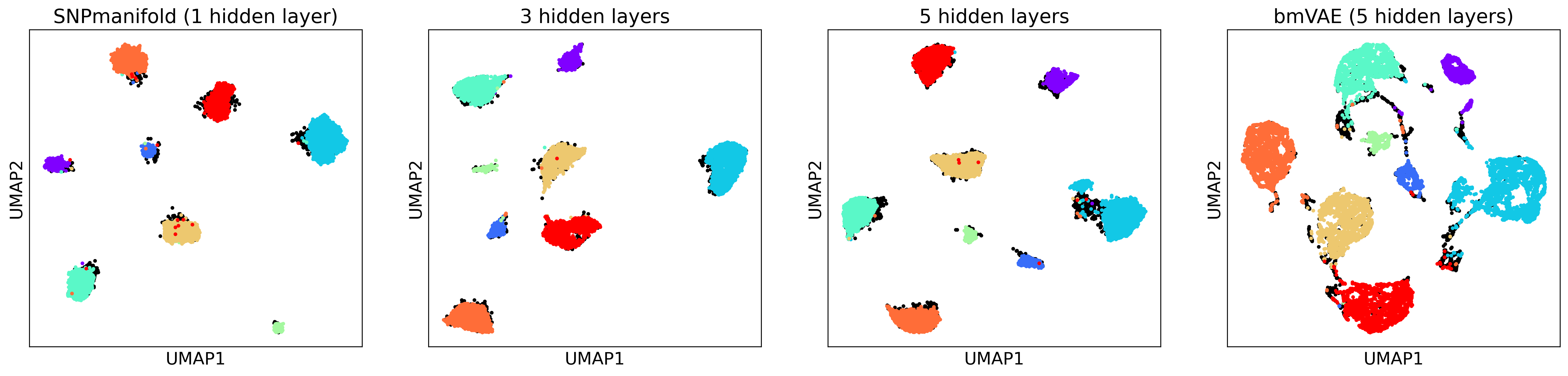

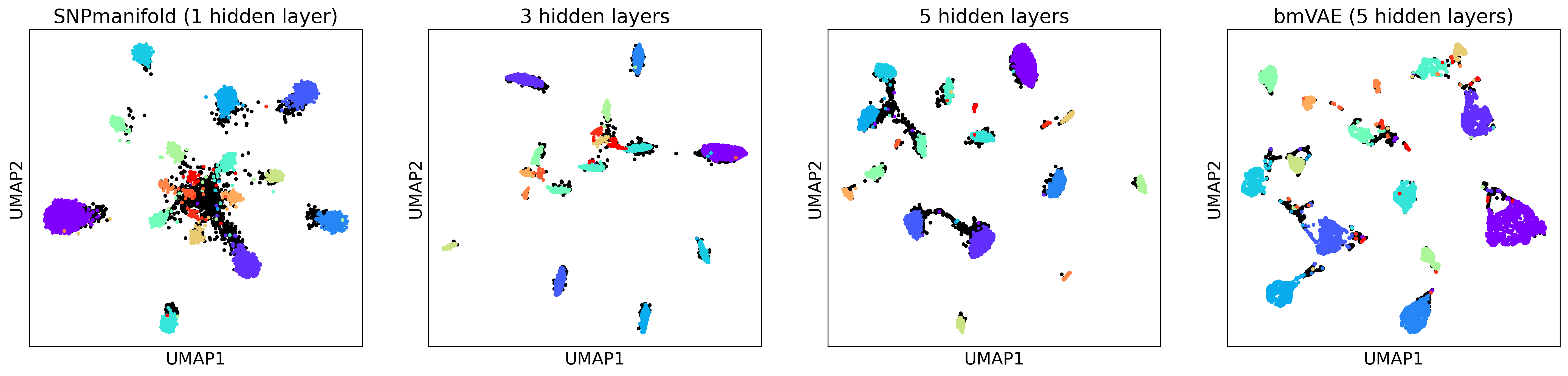


**Supp. Fig. S2.** SNV embedding manifolds of Donor4, Donor8, and Donor18 datasets from up to down, colored by donors (ground truth) in VAEs with 1 hidden layer (SNPmanifold), 3 hidden layers, 5 hidden layers, and bmVAE hyperparameters (5 hidden layers, D = 3, β = 0.0001). Black dots represent doublets. VAE with 1 hidden layer separates different donors better than deeper VAEs with 3 hidden layers, 5 hidden layers, and bmVAE hyperparameters.


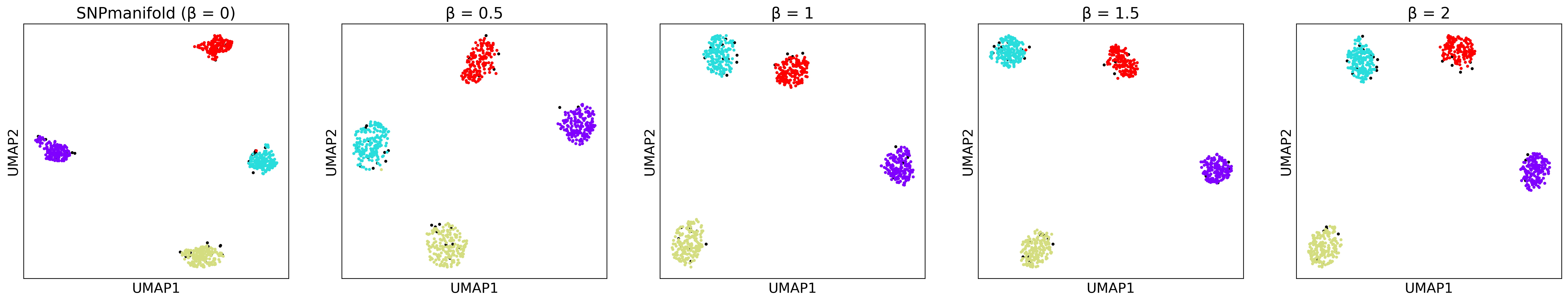

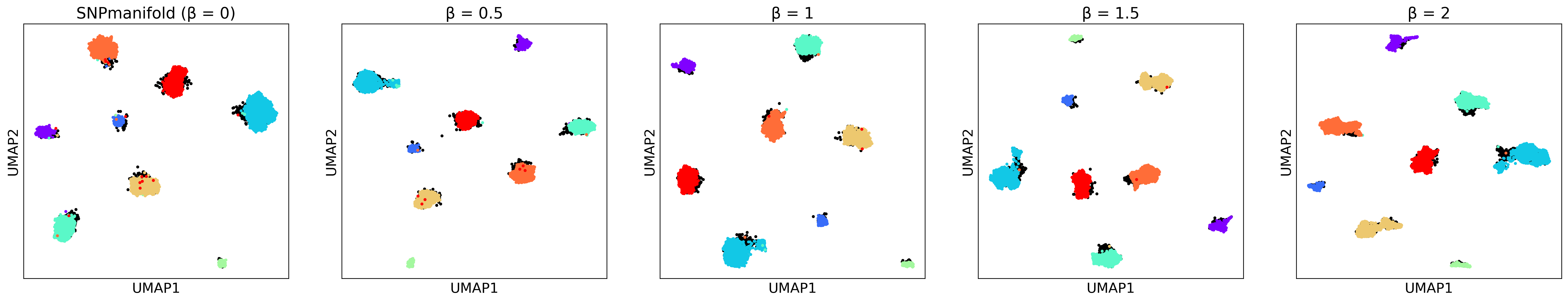

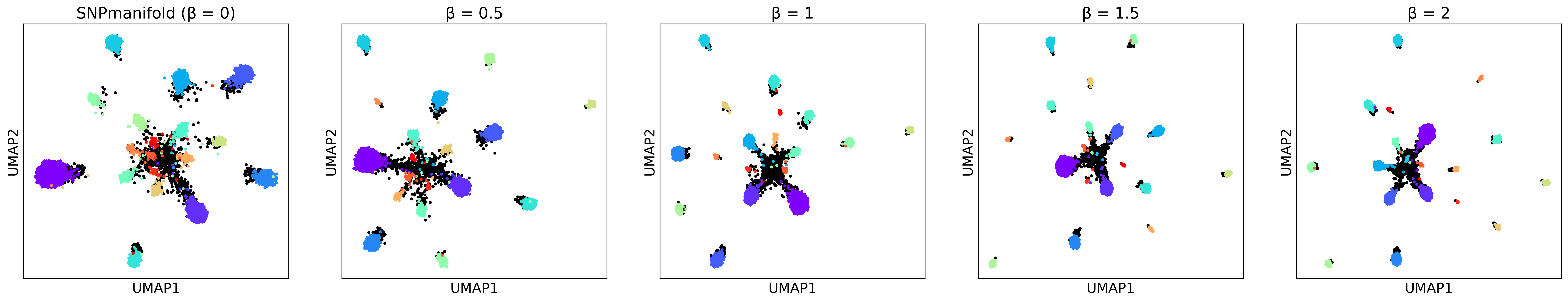


**Supp. Fig. S3.** SNV embedding manifolds of Donor4, Donor8, and Donor18 datasets from up to down, colored by donors (ground truth) in VAEs with β = 0 (SNPmanifold), 0.5, 1, 1.5, 2. Black dots represent doublets. VAEs with different values of β separates different donors similarly.


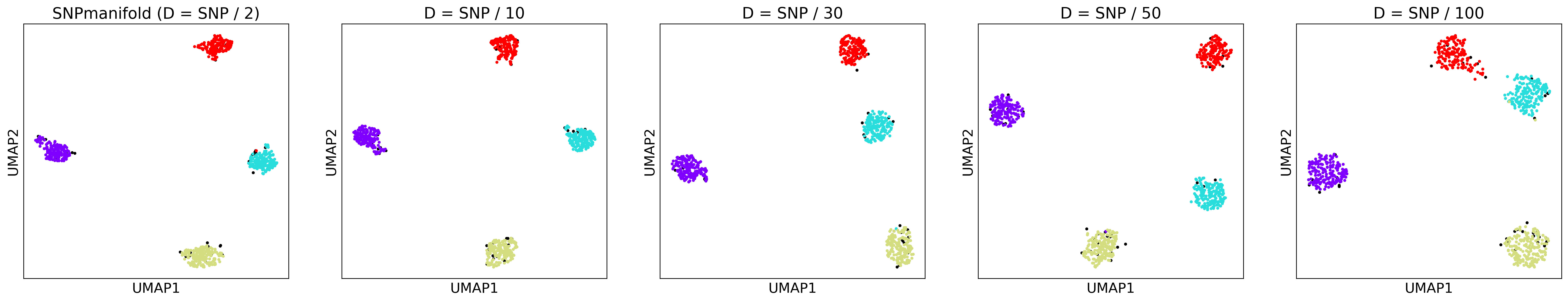

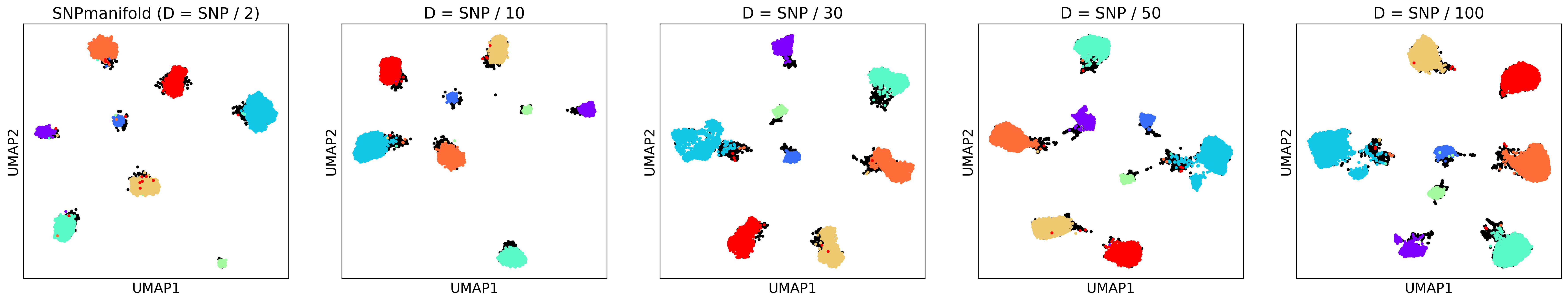

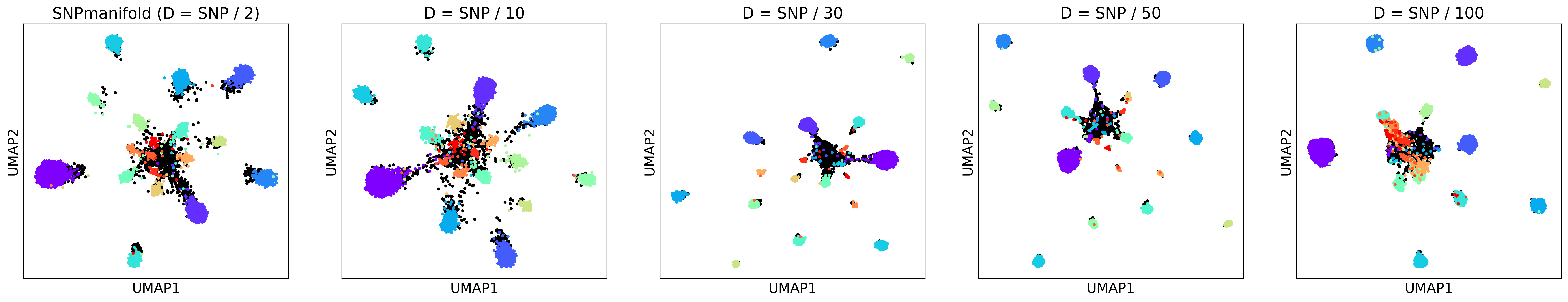


**Supp. Fig. S4.** SNV embedding manifolds of Donor4, Donor8, and Donor18 datasets from up to down, colored by donors (ground truth) in VAEs with numbers of latent dimensions D = no. of SNPs / (2 (SNPmanifold), 10, 30, 50, 100). Black dots represent doublets. VAEs with higher values of D = (no. of SNPs / (2, 10)) separate different donors better than VAEs with lower values of D = (no. of SNPs / (30, 50, 100)).

| **Dataset** | **Cell type** | **Technology** | **No. of cells** | **No. of SNPs** |
| --- | --- | --- | --- | --- |
| Donor4[19] | Cells from 4 human donors; nuclear | scRNA-seq | 734 | 1905 |
| Donor8[31] | PBMCs from 8 human donors; nuclear | scRNA-seq | 13939 | 929 |
| Donor18[32] | iPSCs from 18 human donors; nuclear | scRNA-seq | 9436 | 864 |
| TF1_GM11906[14] | 2 hematopoietic cell lines; mitochondrial | mtscATAC-seq | 1001 | 56 |
| MKN45[33] | Gastric cancer cell line; mitochondrial | scDNA-seq | 5199 | 322 |
| BPDCN[9] | PBMCs from 1 human donor; mitochondrial | MAESTER (mtscRNA-seq) | 9204 | 274 |
| HSPC_PBMC[14] | HSPCs and PBMCs from 1 human donor; mitochondrial | mtscATAC-seq | 10936 | 100 |

**Supp. Fig. S5.** Datasets used in this work. There are 3 donor-multiplexing datasets with germline nuclear SNPs (Donor4, Donor8, Donor18) and 4 somatic lineage tracing datasets with mitochondrial SNPs (TF1_GM11906, MKN15, BPDCN, HSPC_PBMC). Machine-readable xlsx format can be found in Supp. Table S2.


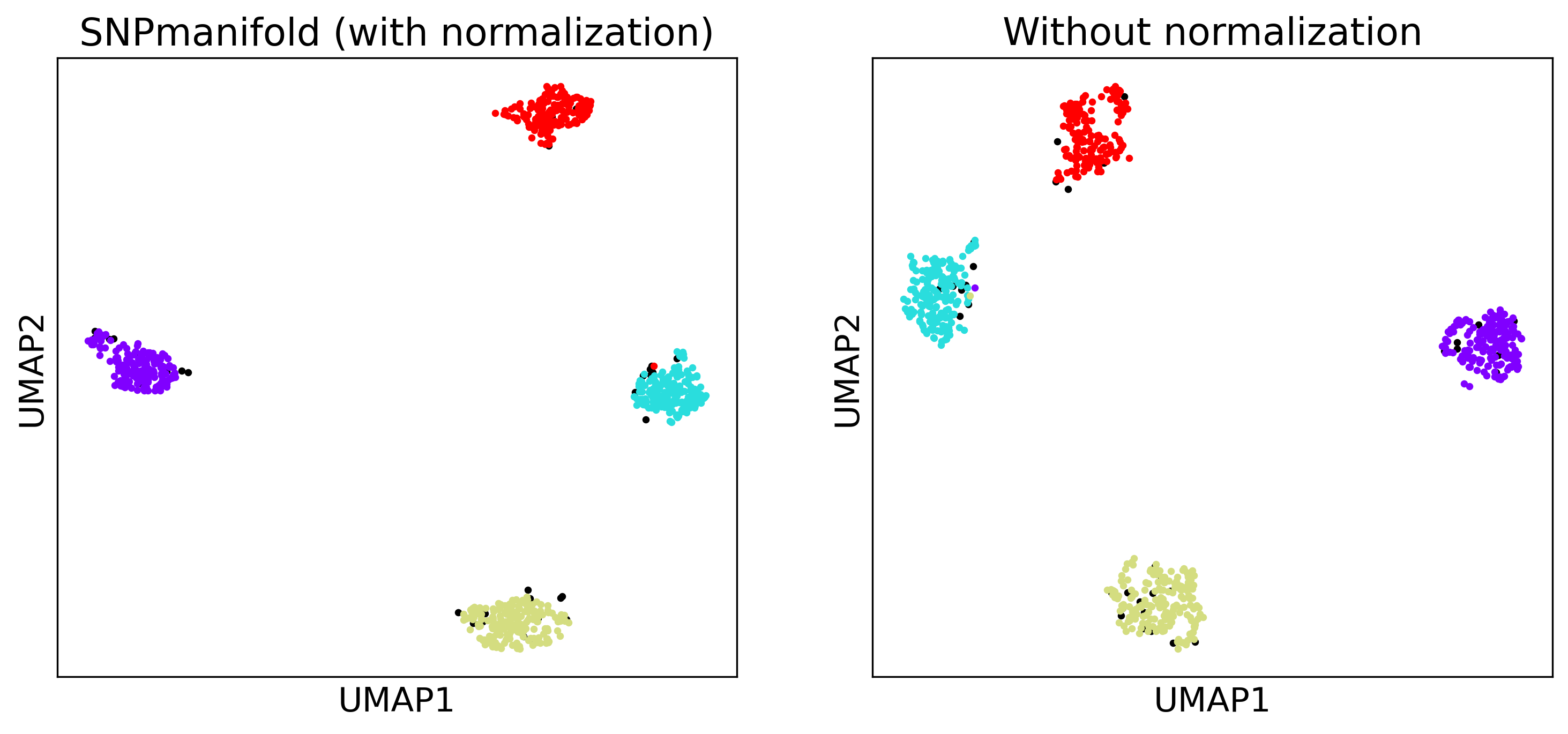

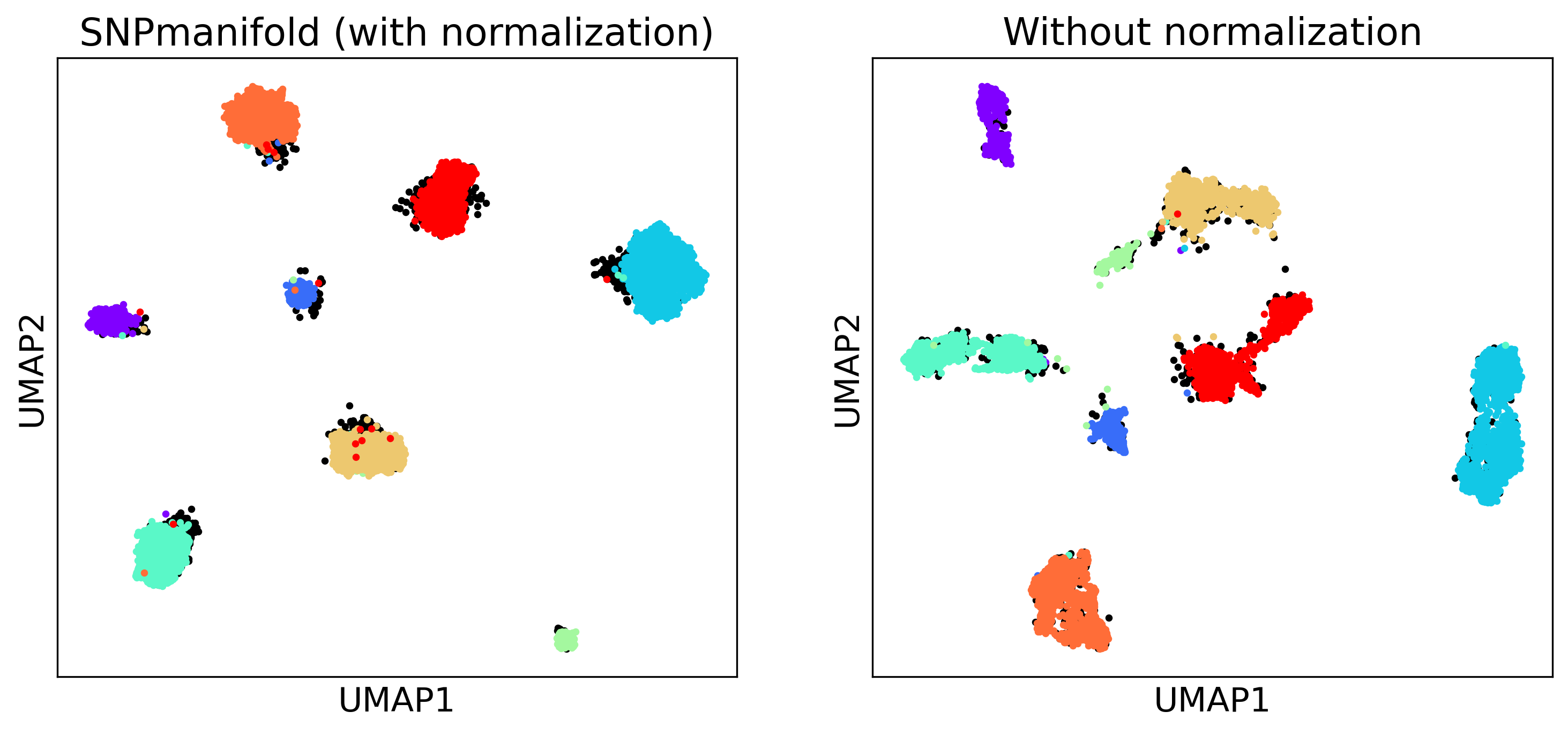

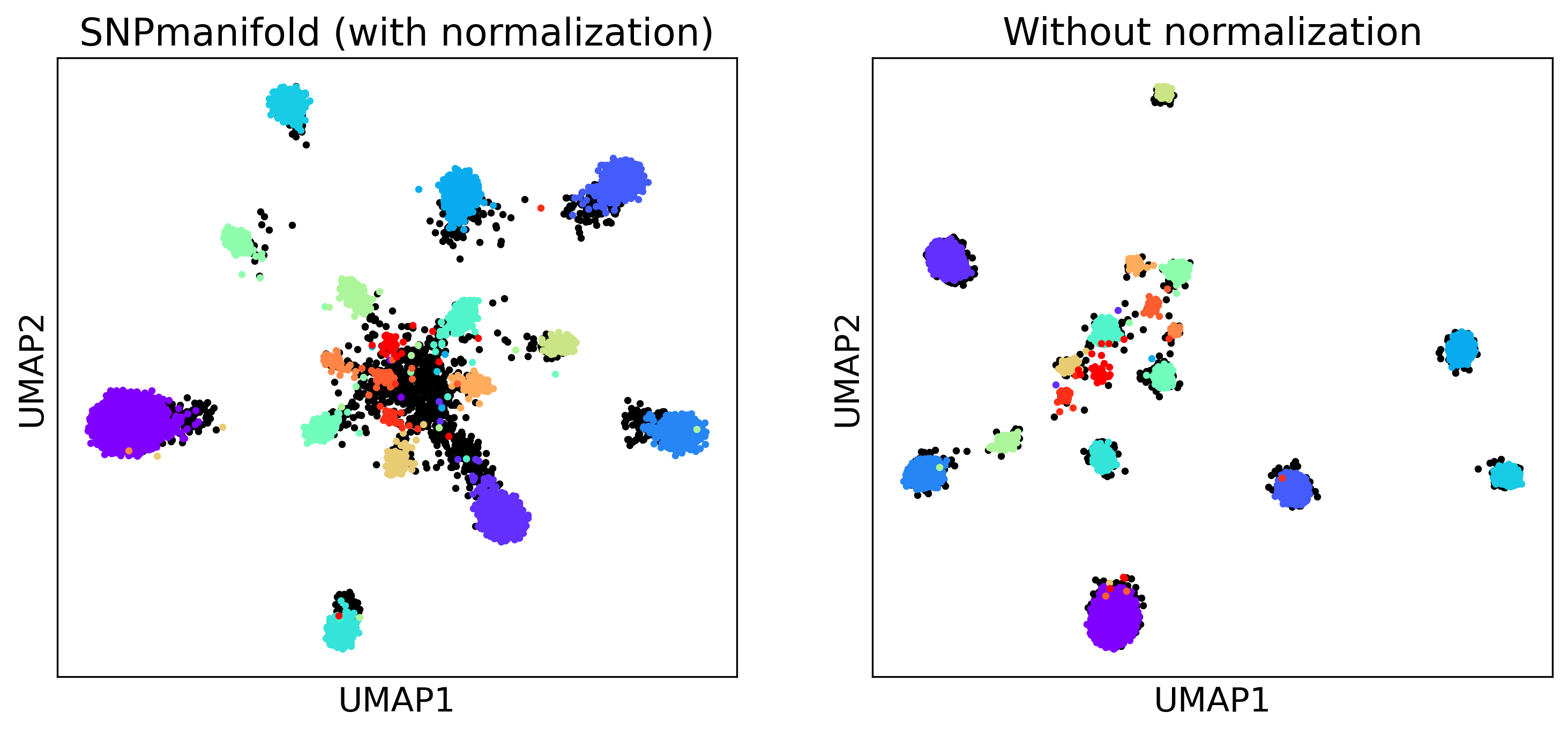


**Supp. Fig. S6.** SNV embedding manifolds of Donor4, Donor8, and Donor18 datasets from up to down, colored by donors (ground truth) in VAEs with or without ‘observed-SNP normalization’. Black dots represent doublets. VAE with ‘observed-SNP normalization’ separates different donors better than VAE without ‘observed SNP normalization’, especially in Donor8 dataset, a dataset with higher cell type diversity (peripheral blood mononuclear cells).


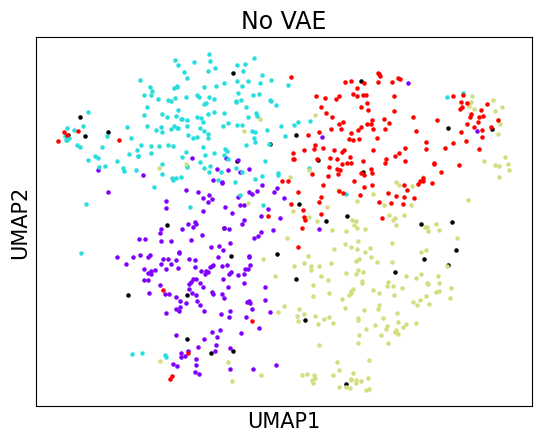

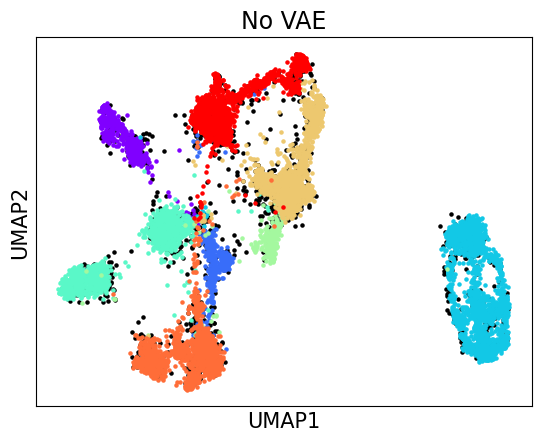

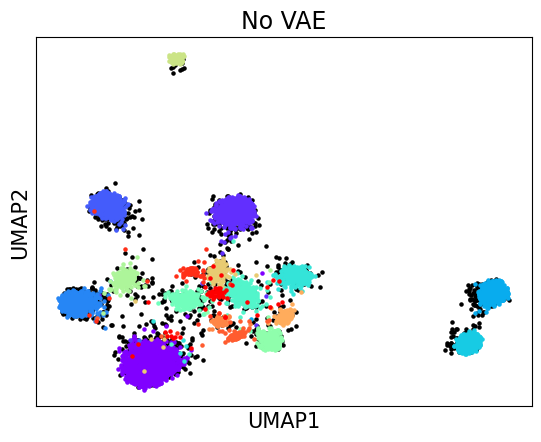

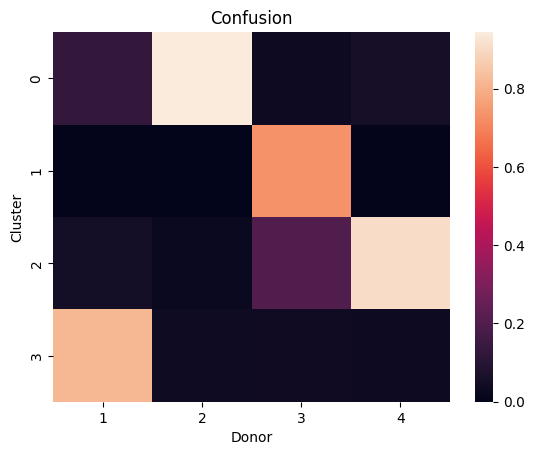

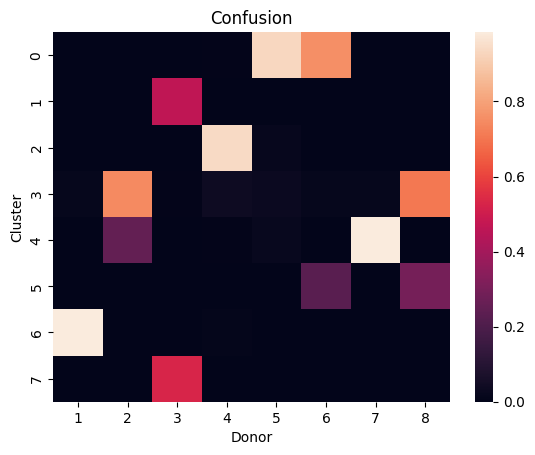

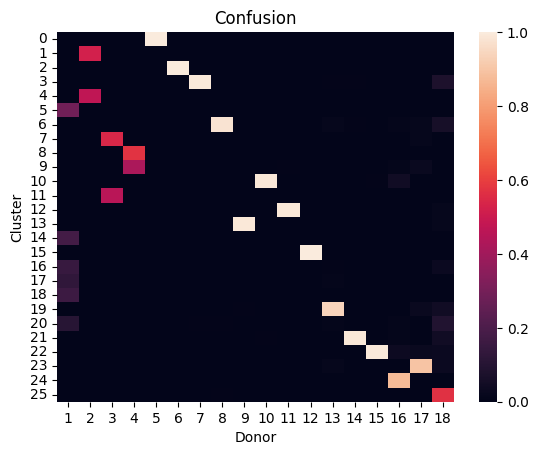


**Supp. Fig. S7.** SNV embedding manifolds of UMAP with no VAE and confusion matrices between donor labels and cluster labels of Donor4, Donor8, and Donor18 datasets from left to right, colored by donors (ground truth). Black dots represent doublets. Accuracy and silhouette score of UMAP alone are not as good as SNPmanifold.


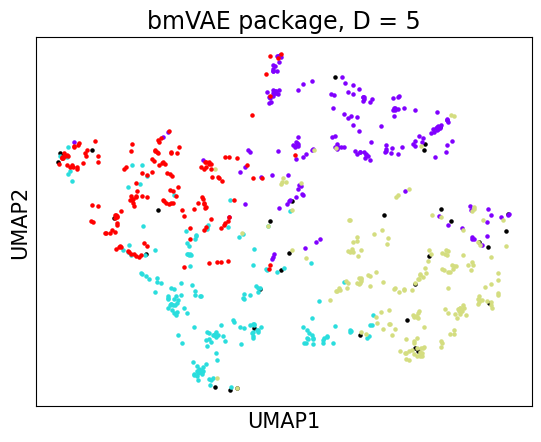

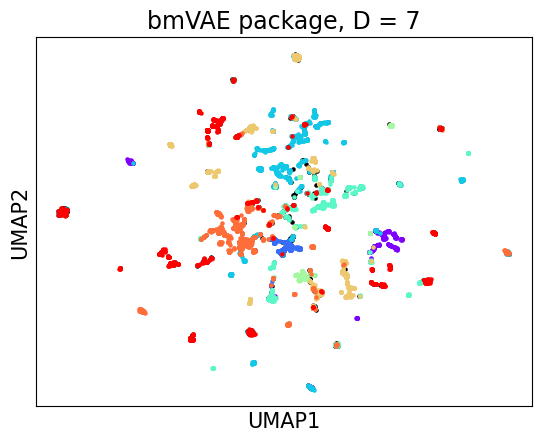

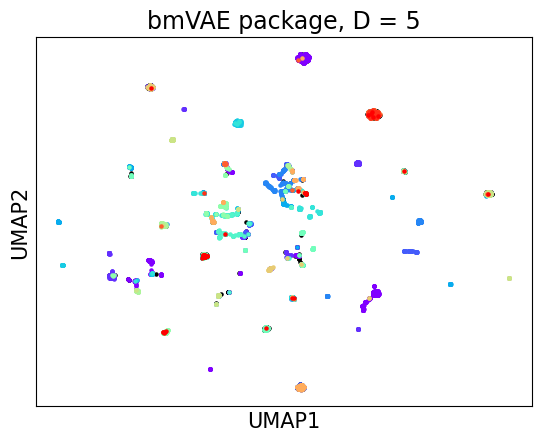

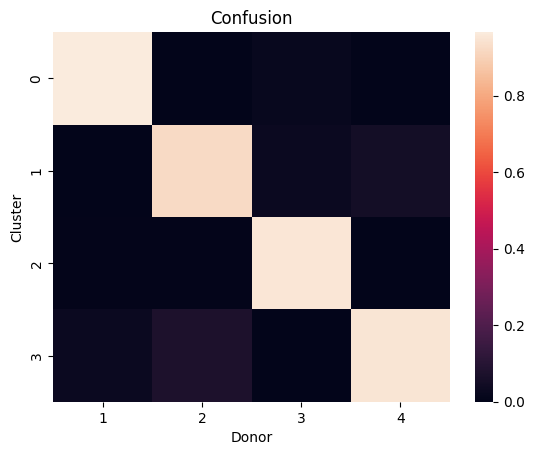

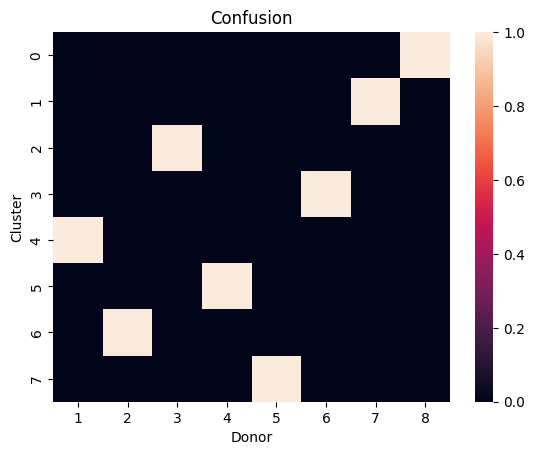

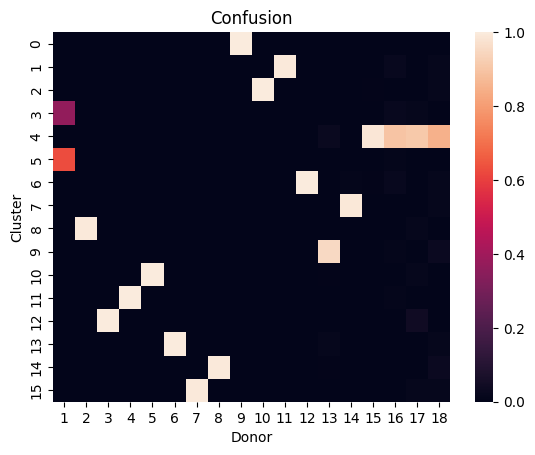


**Supp. Fig. S8.** SNV embedding manifolds of bmVAE package and confusion matrices between donor labels and cluster labels of Donor4, Donor8, and Donor18 datasets from left to right, colored by donors (ground truth). Black dots represent doublets. We tested bmVAE package with different numbers of latent dimensions (D = 3, 5, 7,10) and picked the best performance for comparison. Since bmVAE package does not support setting of the exact number of clusters, the number of clusters may not be equal to the number of donors. In terms of accuracy, bmVAE package is comparable to SNPmanifold in Donor8 dataset, but worse than SNPmanifold in Donor4 and Donor18 dataset. In terms of silhouette score, bmVAE package is significantly worse than SNPmanifold in all 3 datasets. This directly results in the messy visualization of SNV embedding under UMAP.


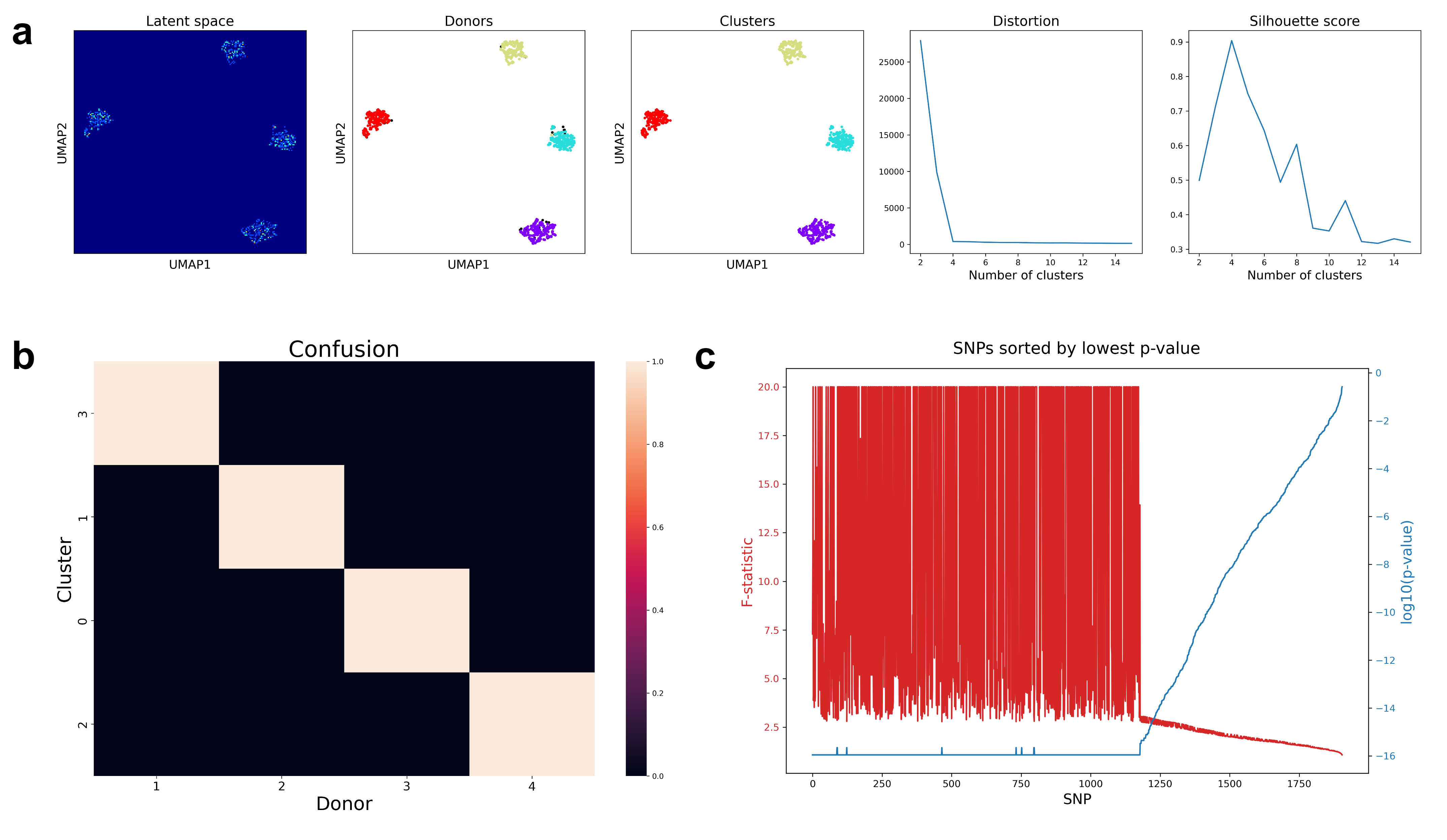


**Supp. Fig. S9.** **a,** Density plot of SNV embedding manifold of Donor4 dataset, donor labels and cluster labels on the manifold, distortion and silhouette scores of k-means clustering. There are 4 disconnected manifolds in the density plot corresponding to 4 donors, and k-means clustering identifies them successfully with good clustering metrics. **b,** Confusion matrix between donor labels and cluster labels. The accuracy reaches 99.8%. **c,** F-statistics and p-values of the SNPs ranked by SNPmanifold, SNPs at left are considered more informative.


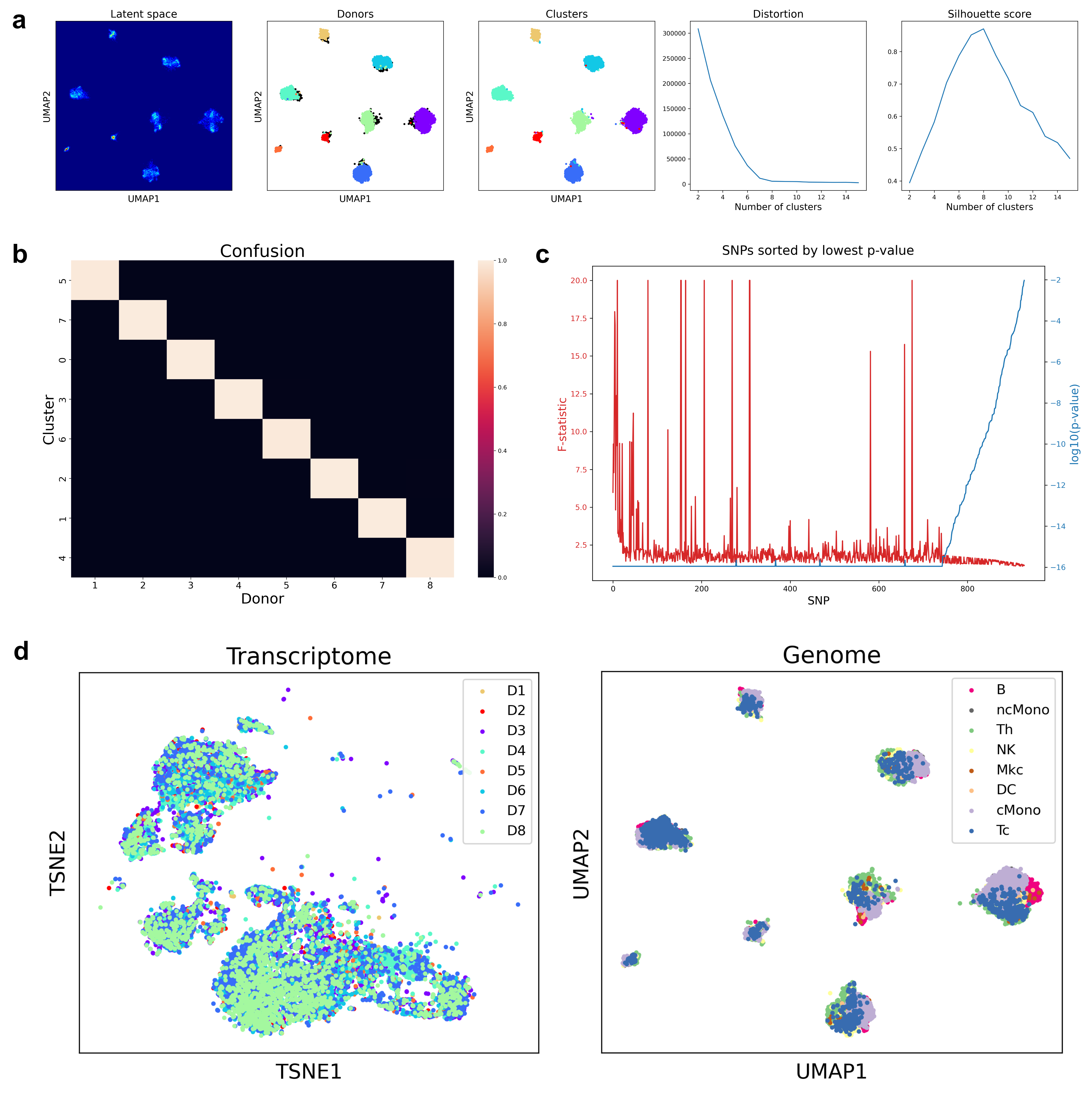


**Supp. Fig. S10.** **a,** Density plot of SNV embedding manifold of Donor8 dataset, donor labels and cluster labels on the manifold, distortion and silhouette scores of k-means clustering. There are 8 disconnected manifolds in the density plot corresponding to 8 donors, and k-means clustering identifies them successfully with good clustering metrics. **b,** Confusion matrix between donor labels and cluster label. The accuracy reaches 99.7%. **c,** F-statistics and p-values of the SNPs ranked by SNPmanifold, SNPs at left are considered more informative. **d,** Cluster labels on transcriptomic t-SNE and cell-type labels on SNV embedding manifold. These two manifolds are largely independent of each other, meaning that they can offer biological insights from different perspectives.


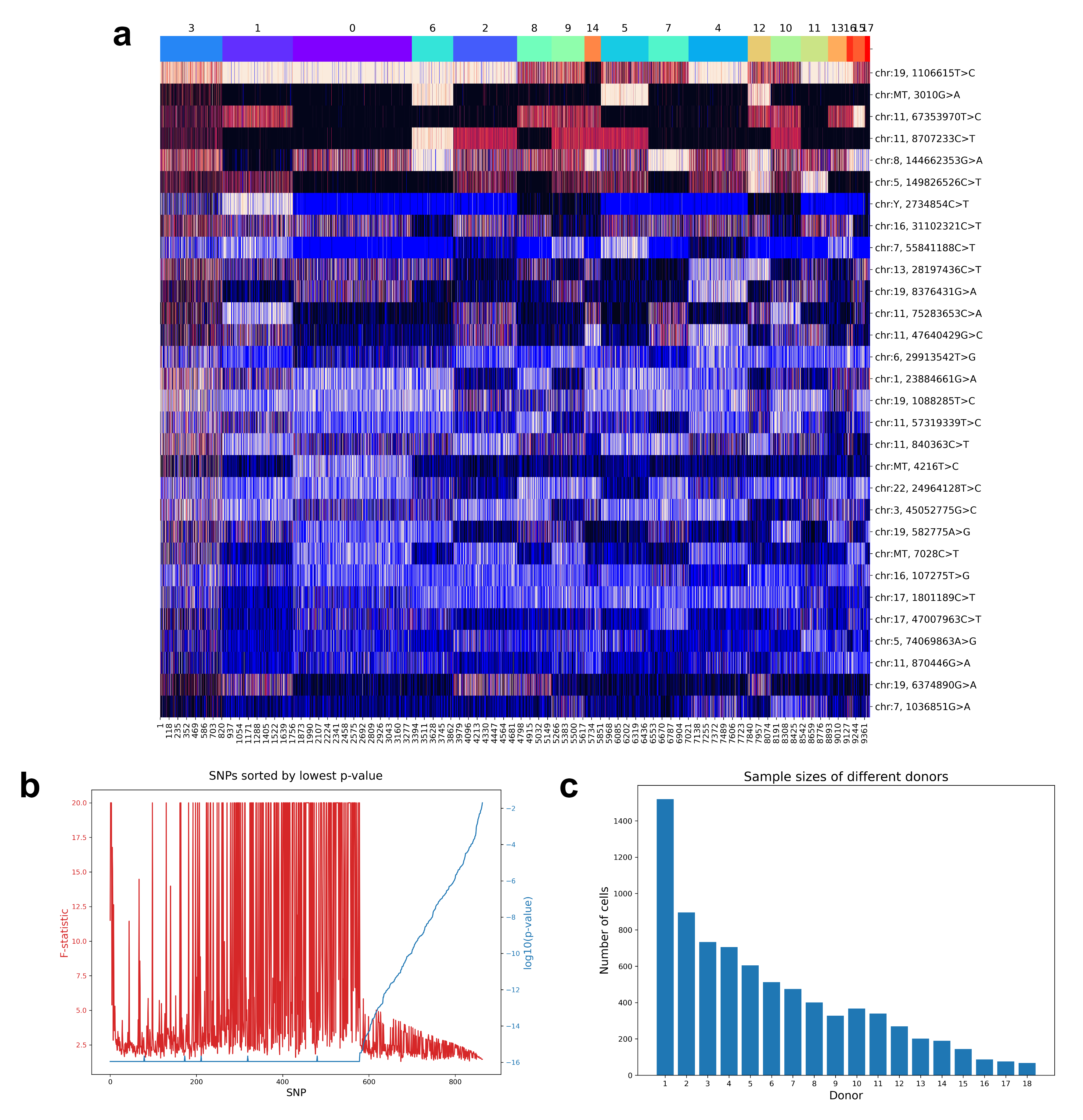


**Supp. Fig. S11.** **a,** Heatmap of allele frequency matrix of Donor18 dataset. Blue color indicates missing values (i.e. DP = 0). Different clusters have distinct allele expressions. **b,** F-statistics and p-values of the SNPs ranked by SNPmanifold, SNPs at left are considered more informative. **c,** Sample sizes of 18 donors. The sample sizes of one donor are very imbalanced and range from 67 to 1519 cells.


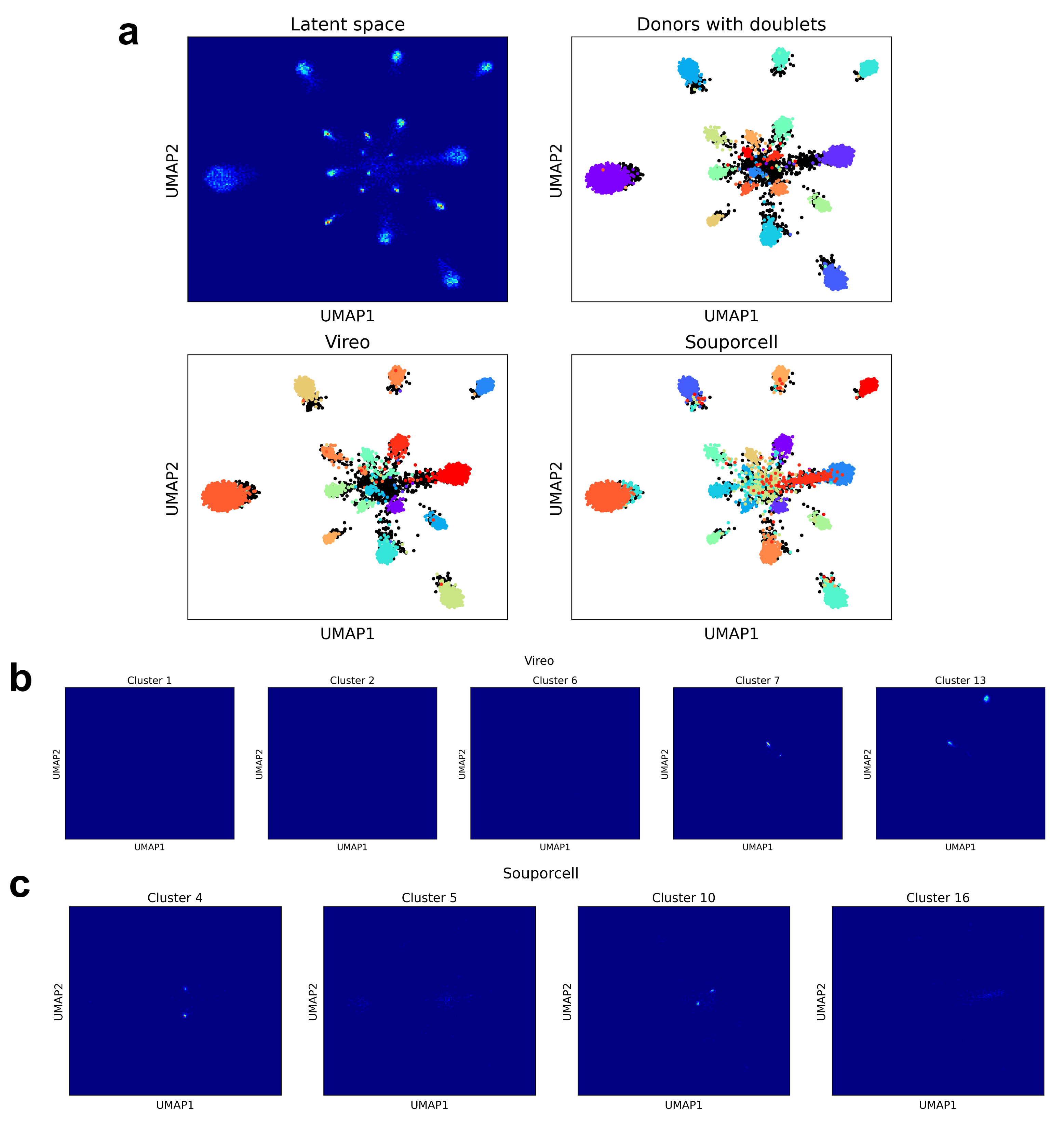


**Supp. Fig. S12.** **a,** Density plot of SNV embedding manifold of Donor18 dataset, donor labels with doublets, Vireo labels and Souporcell labels on the manifold. Vireo without reference to genotypes and Souporcell assigned clusters incorrectly by assigning doublets and combining donors. **b,** Abnormal clusters assigned by Vireo without reference to genotypes. Almost no cells are assigned to clusters 1, 2, 6 while 2 donors are combined together in clusters 7, 13. **c,** Abnormal clusters assigned by Souporcell. Parts of doublets are assigned to clusters 5, 16 while 2 donors are combined together in clusters 4, 10.


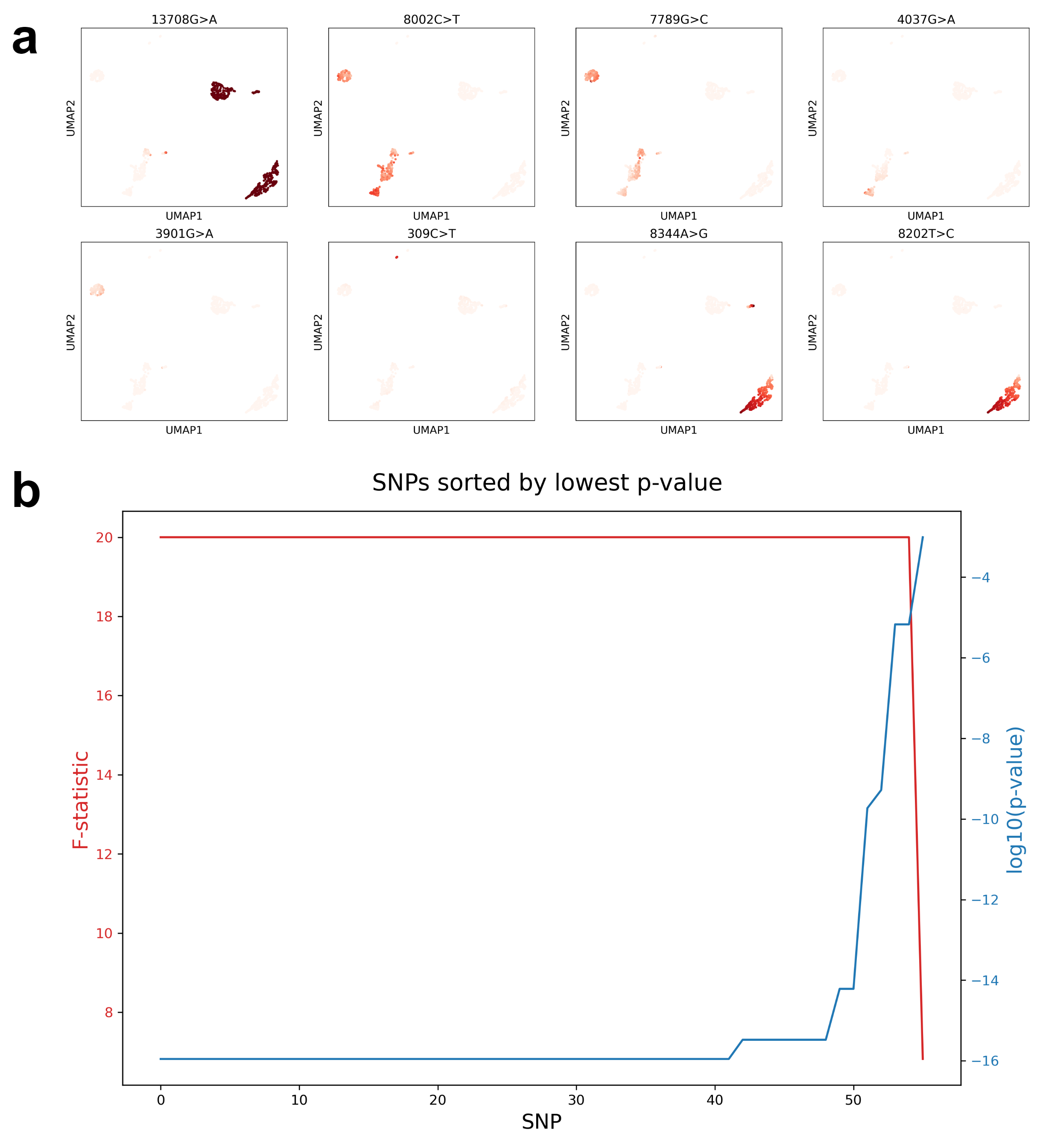


**Supp. Fig. S13.** **a,** Allele frequency of 8 SNPs on SNV embedding manifold of TF1_GM11906 dataset. Different SNPs are localized to different parts of the embedding manifold. **b,** F-statistics and p-values of the SNPs ranked by SNPmanifold, SNPs at left are considered more informative.


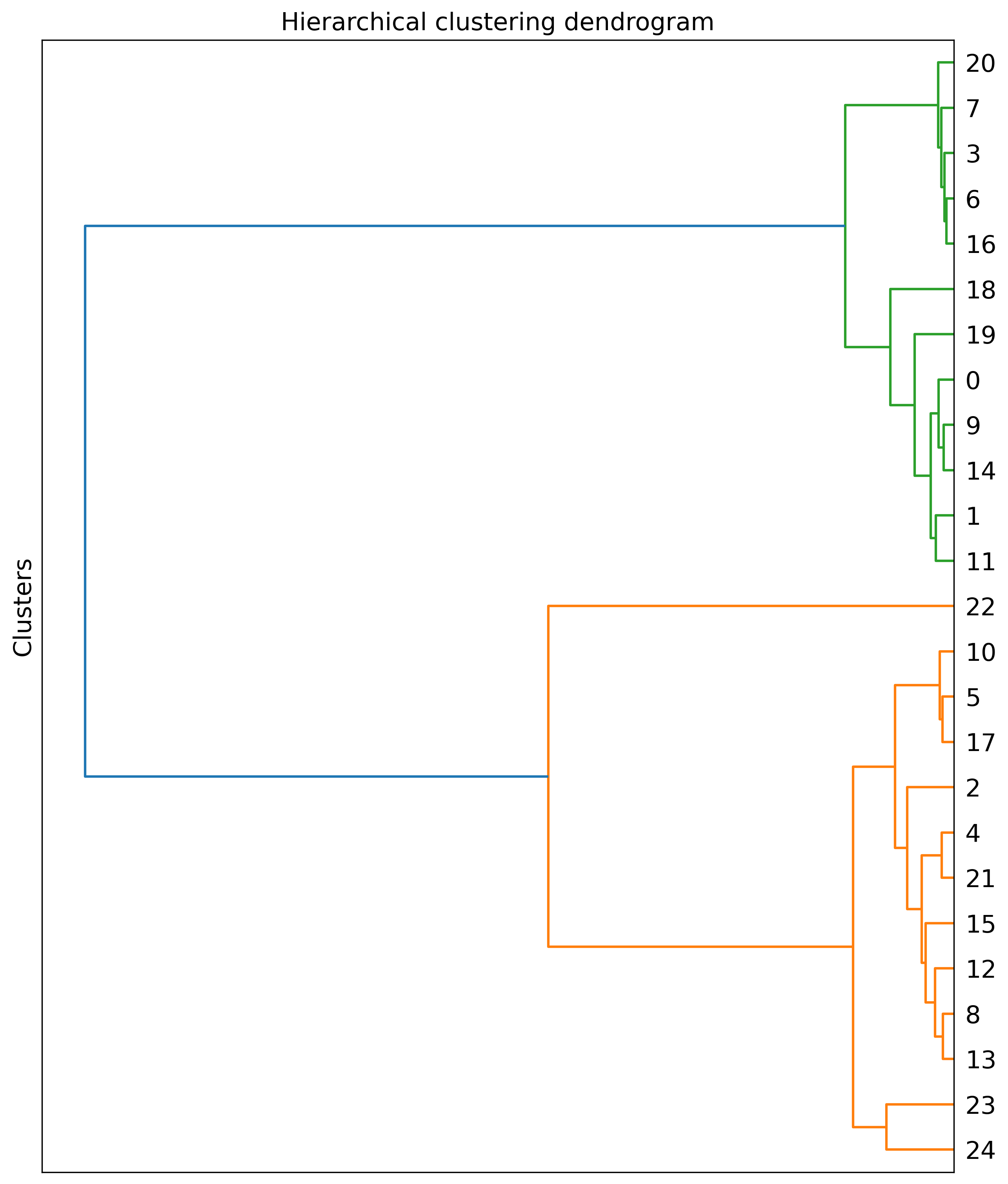


**Supp. Fig. S14.** Hierarchical clustering dendrogram of SNPmanifold clusters in TF1_GM11906 dataset.


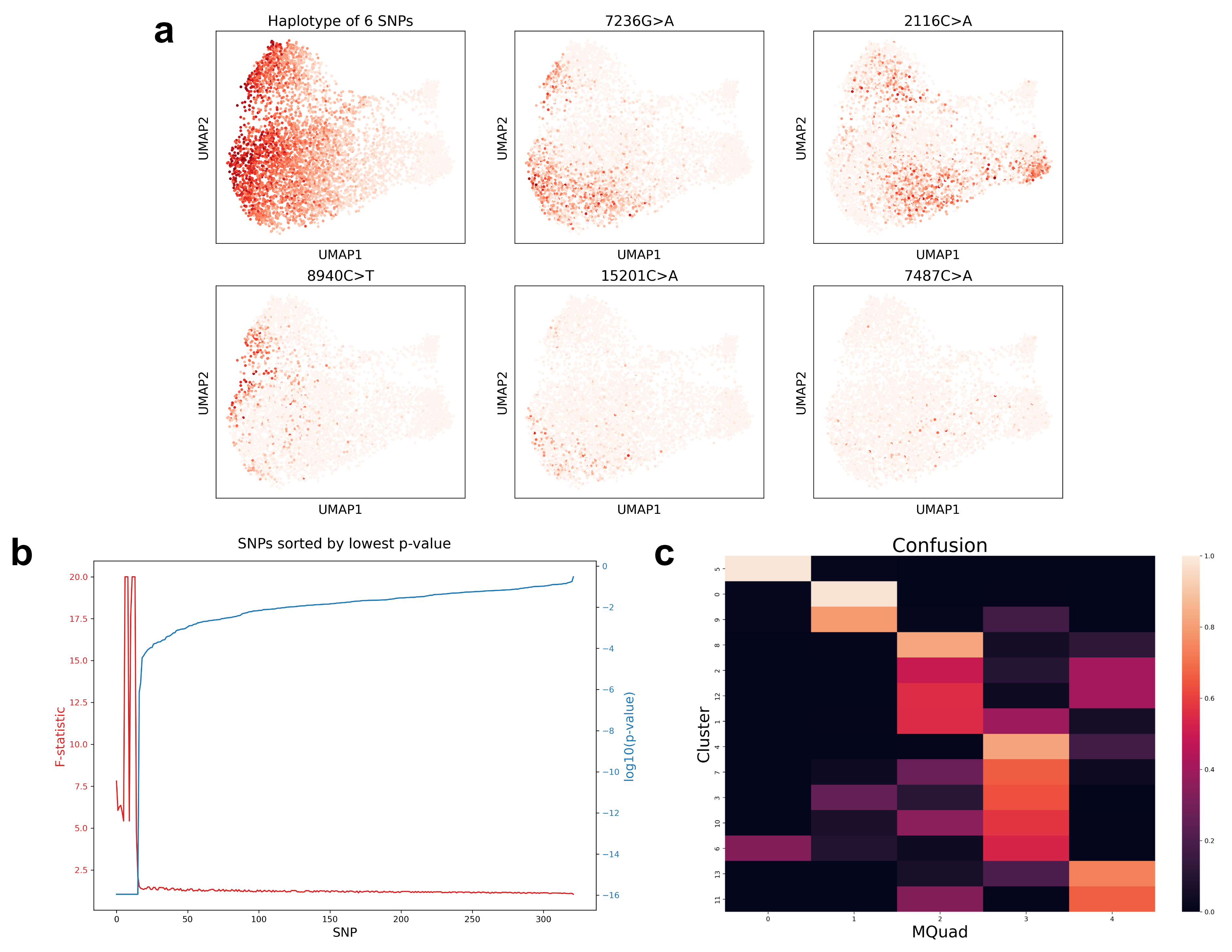


**Supp. Fig. S15.** **a,** Allele frequency of 6 mutations on SNV embedding manifold of MKN45 dataset. Different SNPs are localized to different parts of the embedding manifold. **b,** F-statistics and p-values of the SNPs ranked by SNPmanifold, SNPs at left are considered more informative. **c,** Confusion matrix between MQuad labels and cluster labels. MQuad labels and cluster labels are highly correlated. This suggests that SNPmanifold can achieve higher resolution than MQuad by resolving finer hierarchy of mitochondrial somatic mutations.


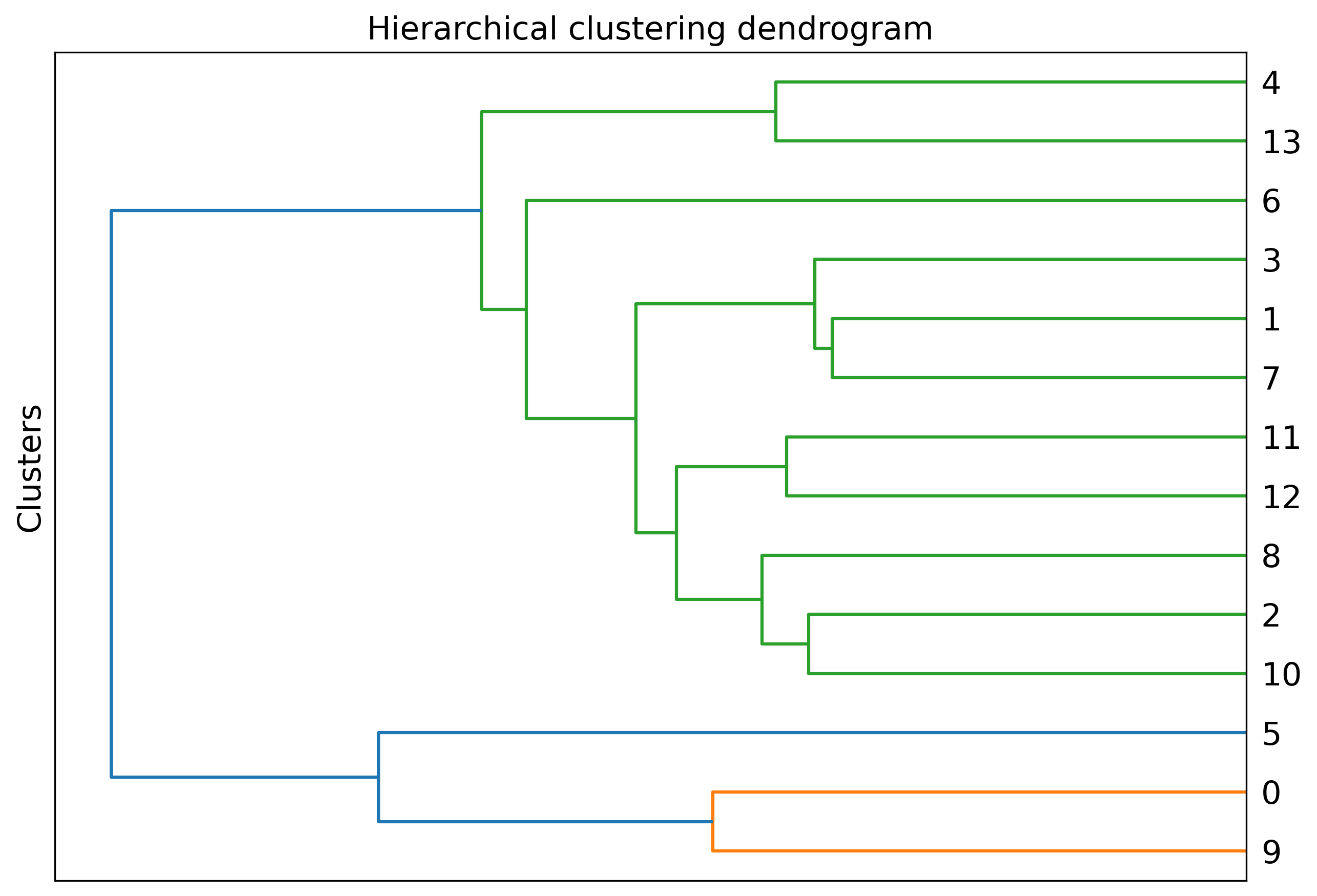


**Supp. Fig. S16.** Hierarchical clustering dendrogram of SNPmanifold clusters in MKN45 dataset.


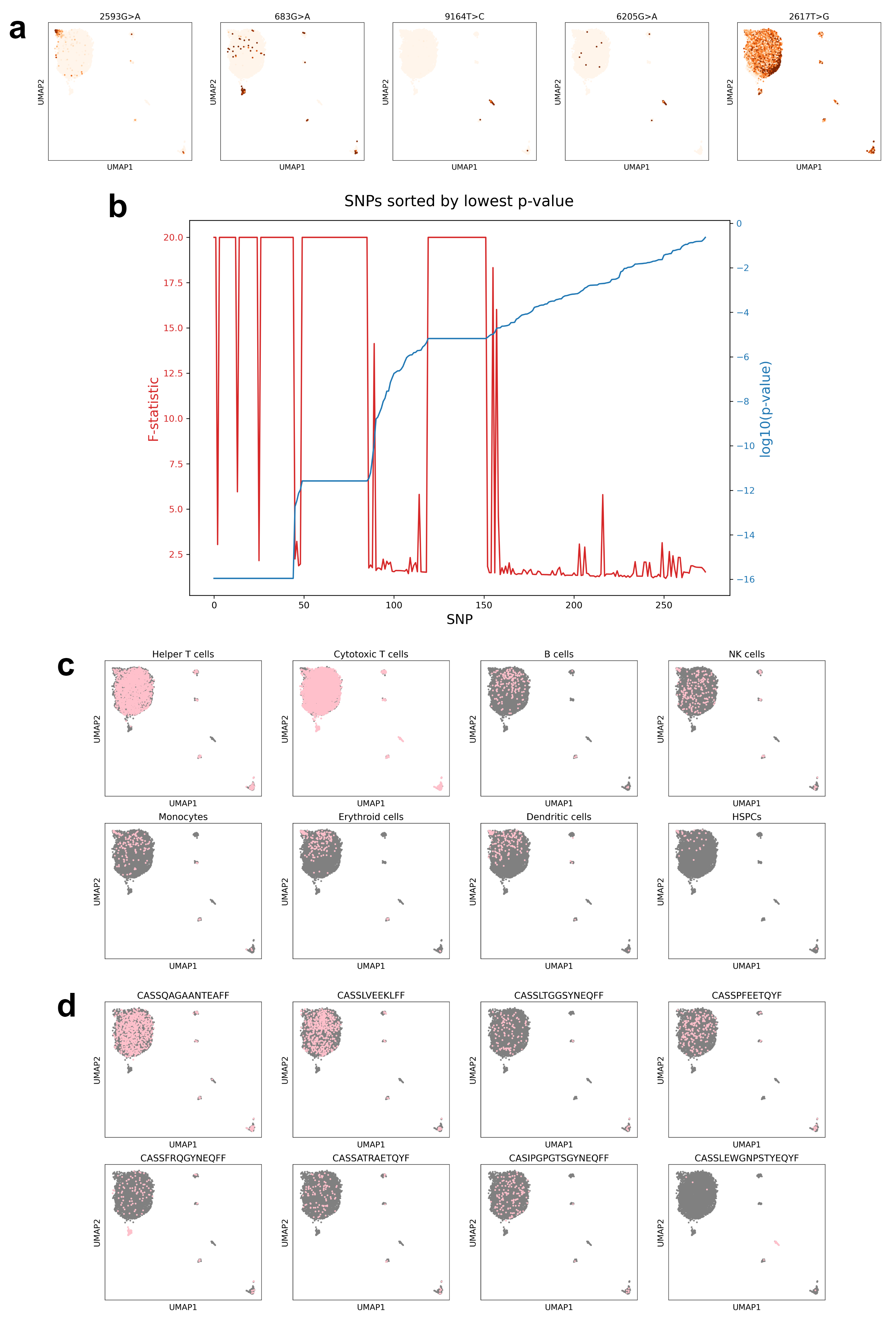


**Supp. Fig. S17.** **a,** Allele frequency of 5 SNPs on SNV embedding manifold of BPDCN dataset. Different SNPs are localized to different parts of the embedding manifold. **b,** F-statistics and p-values of the SNPs ranked by SNPmanifold, SNPs at left are considered more informative. **c,** Cell-type labels on SNV embedding manifold. Different cell types are localized to different parts of the embedding manifold. **d,** TRB-clonotype labels on SNV embedding. Different TRB clonotypes are localized to different parts of the embedding manifold.


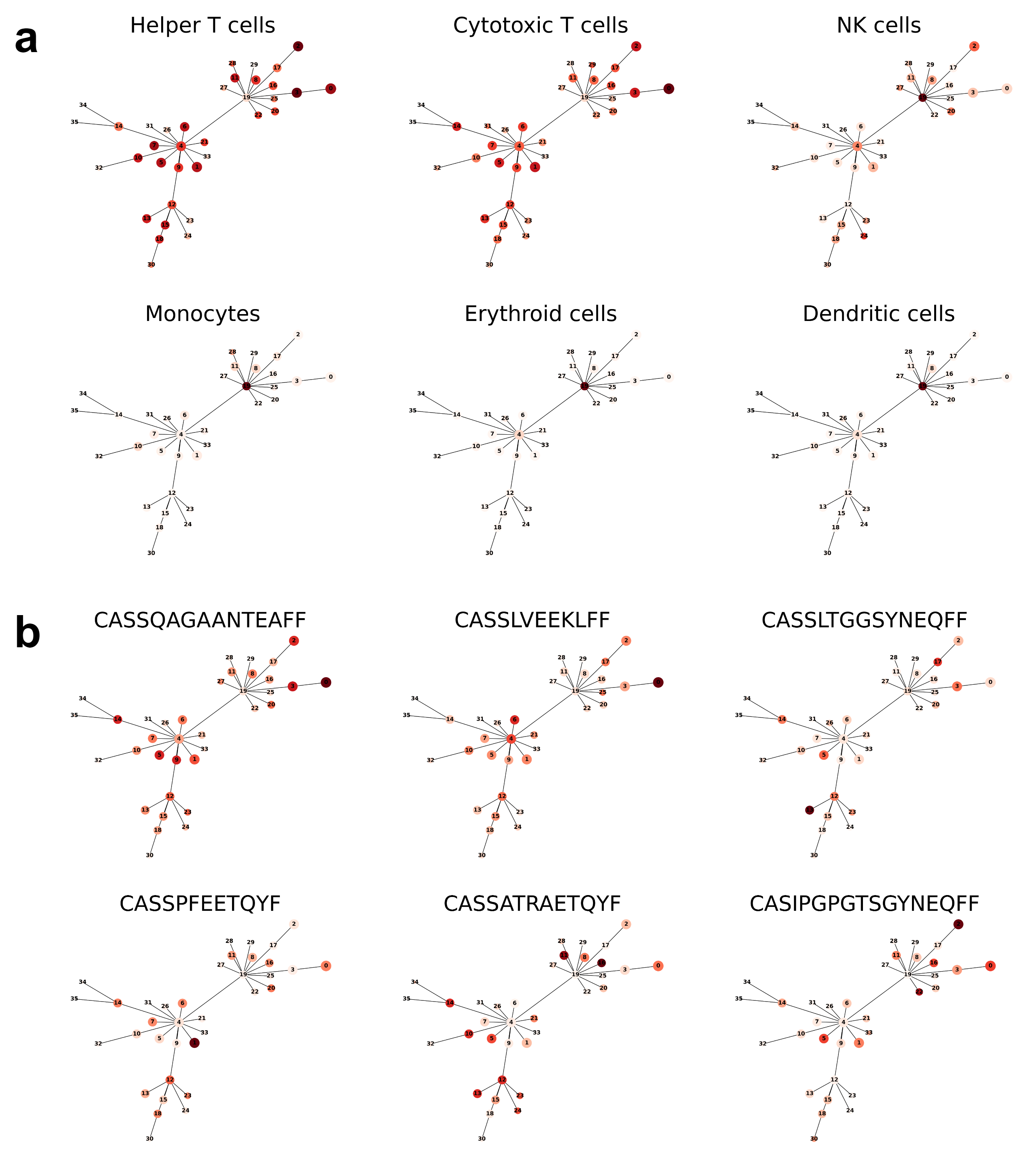


**Supp. Fig. S18.** **a,** Relative abundance of 6 cell types on phylogenetic tree of the manifold of BPDCN dataset. Deeper color indicates higher abundance. There are strong correlations between certain cell types and certain clusters. **b,** Relative abundance of 6 TRB clonotypes on phylogenetic tree of the manifold. Deeper color indicates higher abundance. There are strong correlations between certain TRB clonotypes and certain clusters.


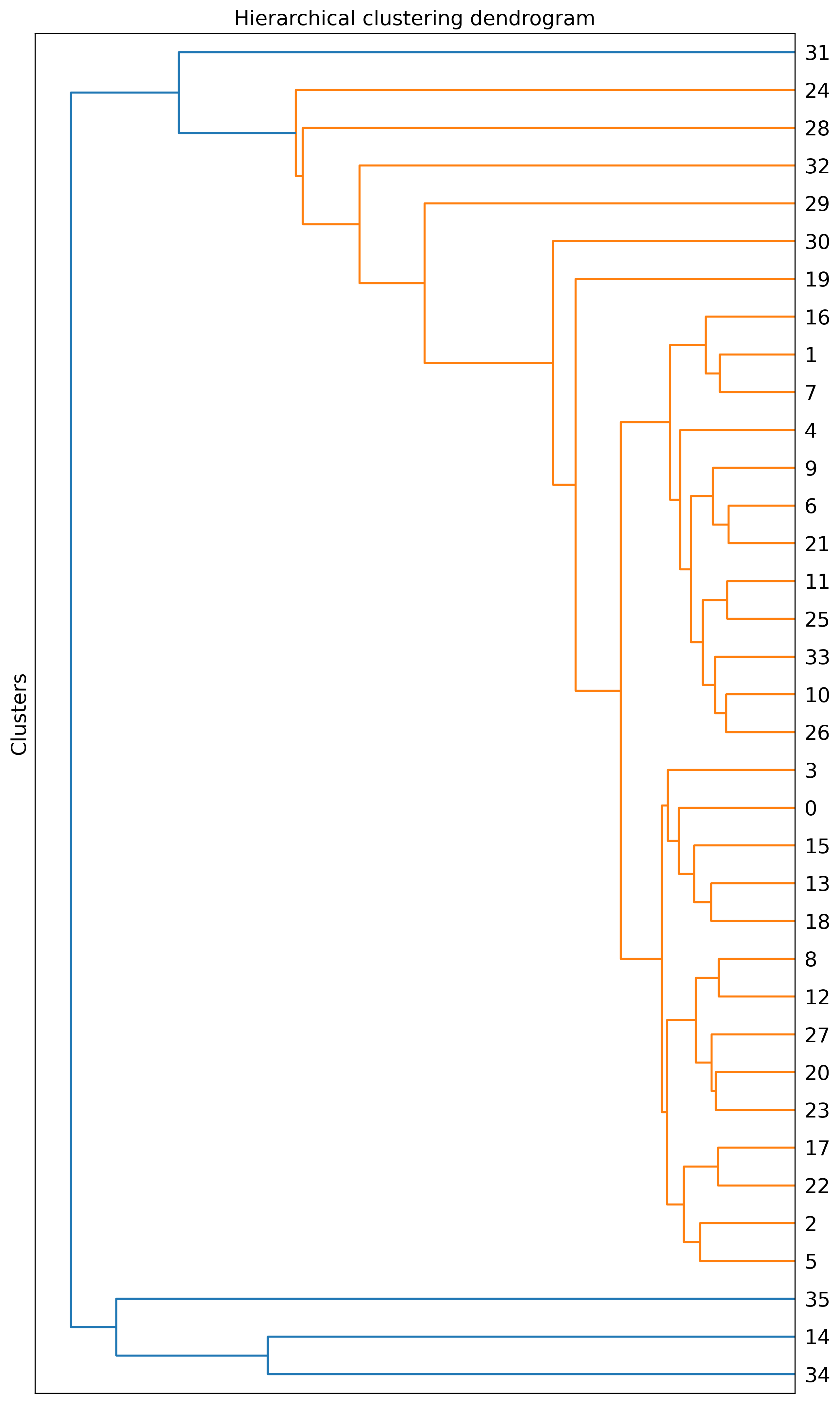


**Supp. Fig. S19.** Hierarchical clustering dendrogram of SNPmanifold clusters in BPDCN dataset.


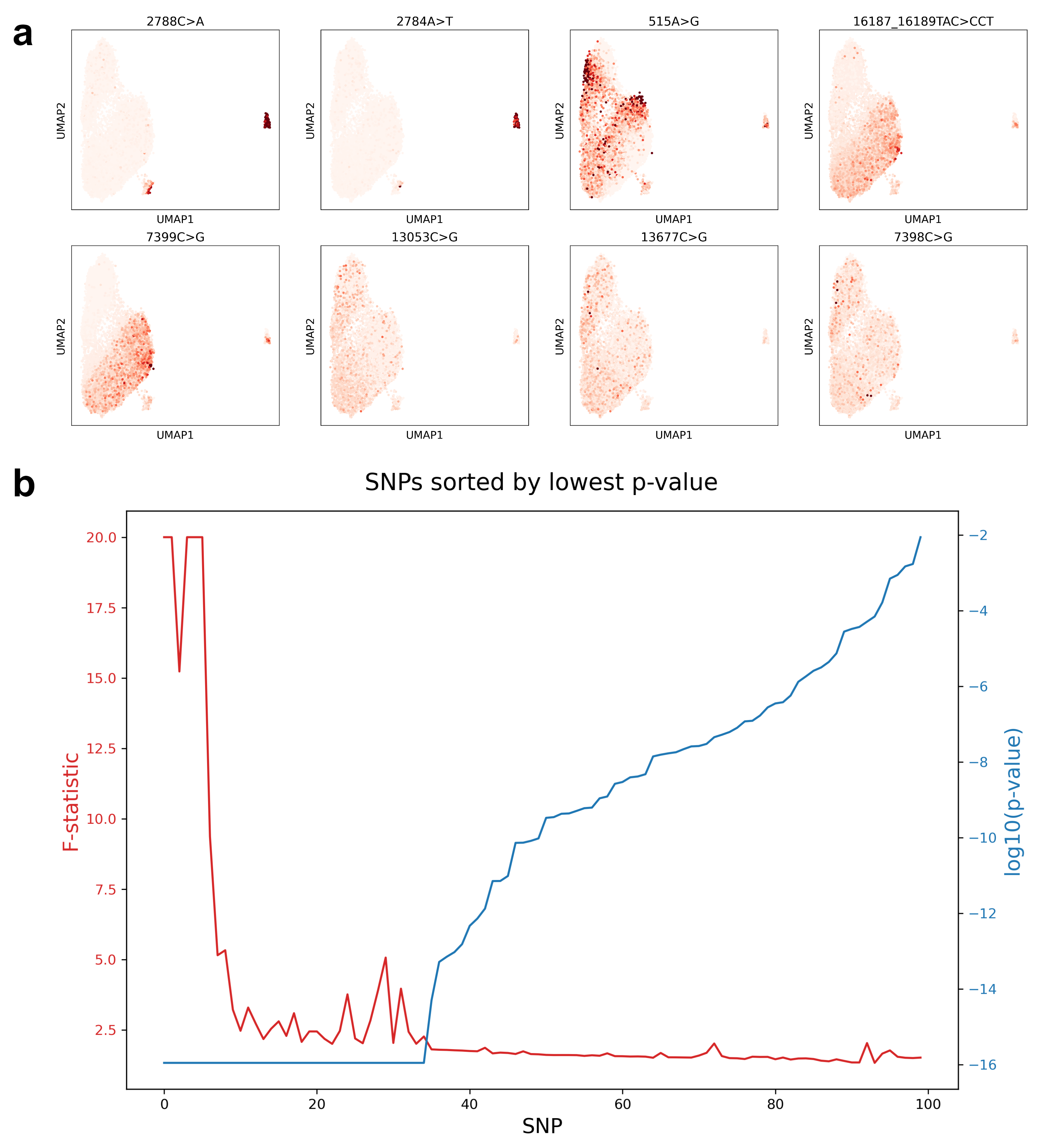


**Supp. Fig. S20.** **a,** Allele frequency of 8 mutations on SNV embedding manifold of HSPC_PBMC dataset. Different SNPs are localized to different parts of the embedding manifold. **b,** F-statistics and p-values of the SNPs ranked by SNPmanifold, SNPs at left are considered more informative.


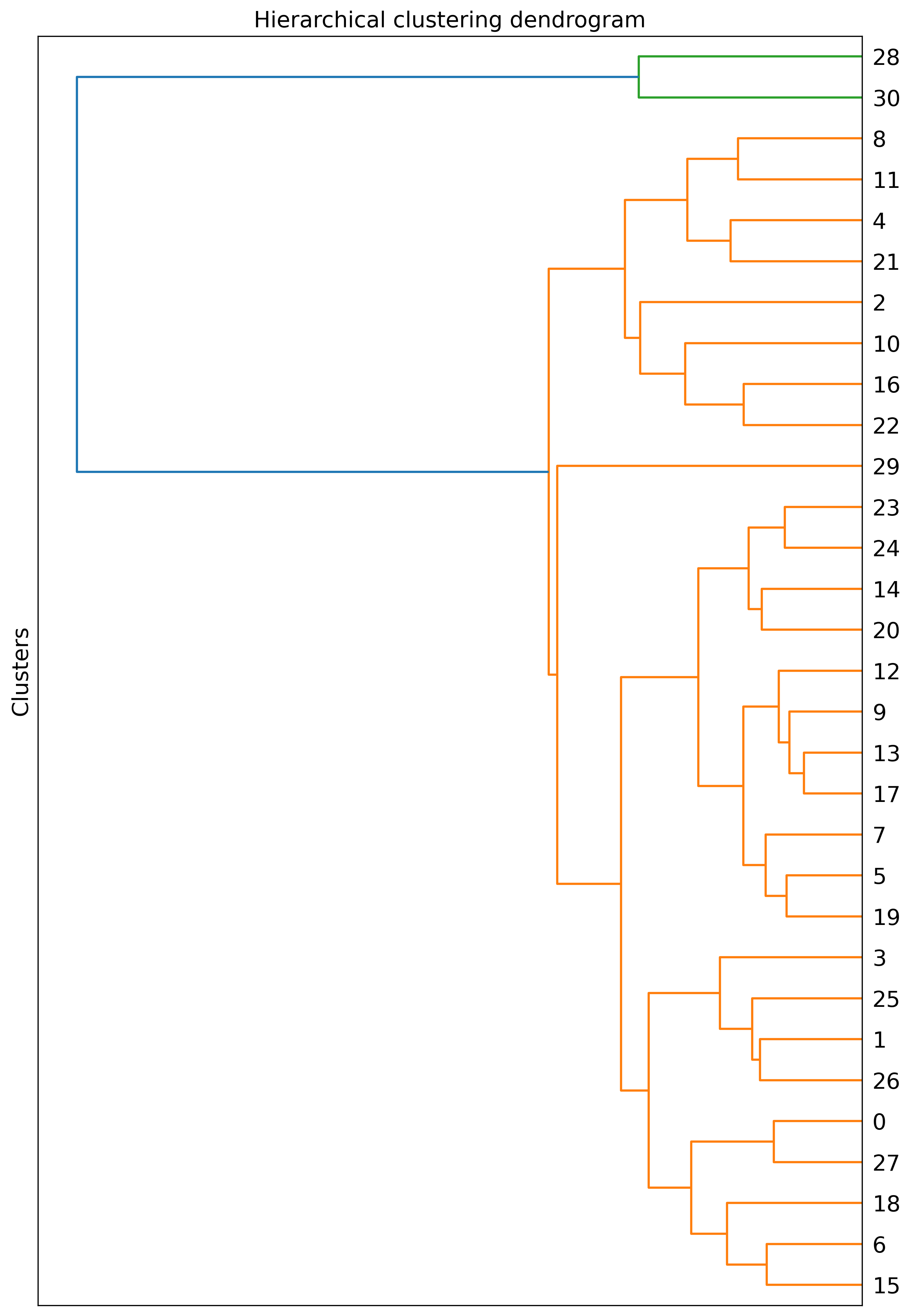


**Supp. Fig. S21.** Hierarchical clustering dendrogram of SNPmanifold clusters in HSPC_PBMC dataset.

| **Dataset** | **cell_SNPread** | **SNP_DPmean** | **SNP_logit_var** |
| --- | --- | --- | --- |
| Donor4 | 50 | 0 | 0.3 |
| Donor8 | 100 | 0 | 0.5 |
| Donor18 | 50 | 0 | 0.3 |
| TF1_GM11906 | 500 | 0 | 0.5 |
| MKN45 | 15000 | 15 | 0.5 |
| BPDCN | 10000 | 5 | 0.5 |
| HSPC_PBMC | 16000 | 0 | 0.5 |

**Supp. Fig. S22.** Filtering thresholds for pre-filtering of different datasets. Machine-readable xlsx format can be found in Supp. Table S3.


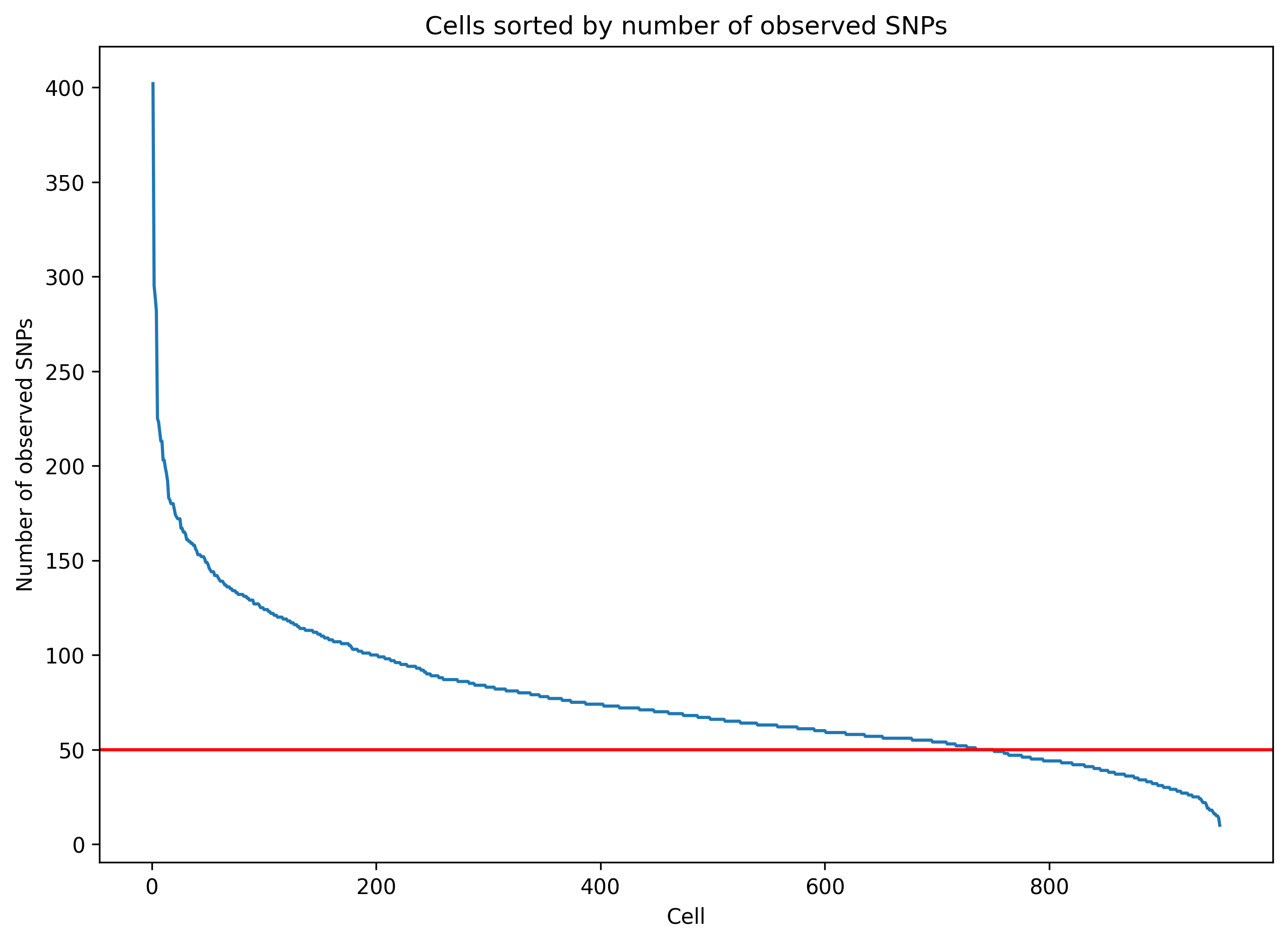

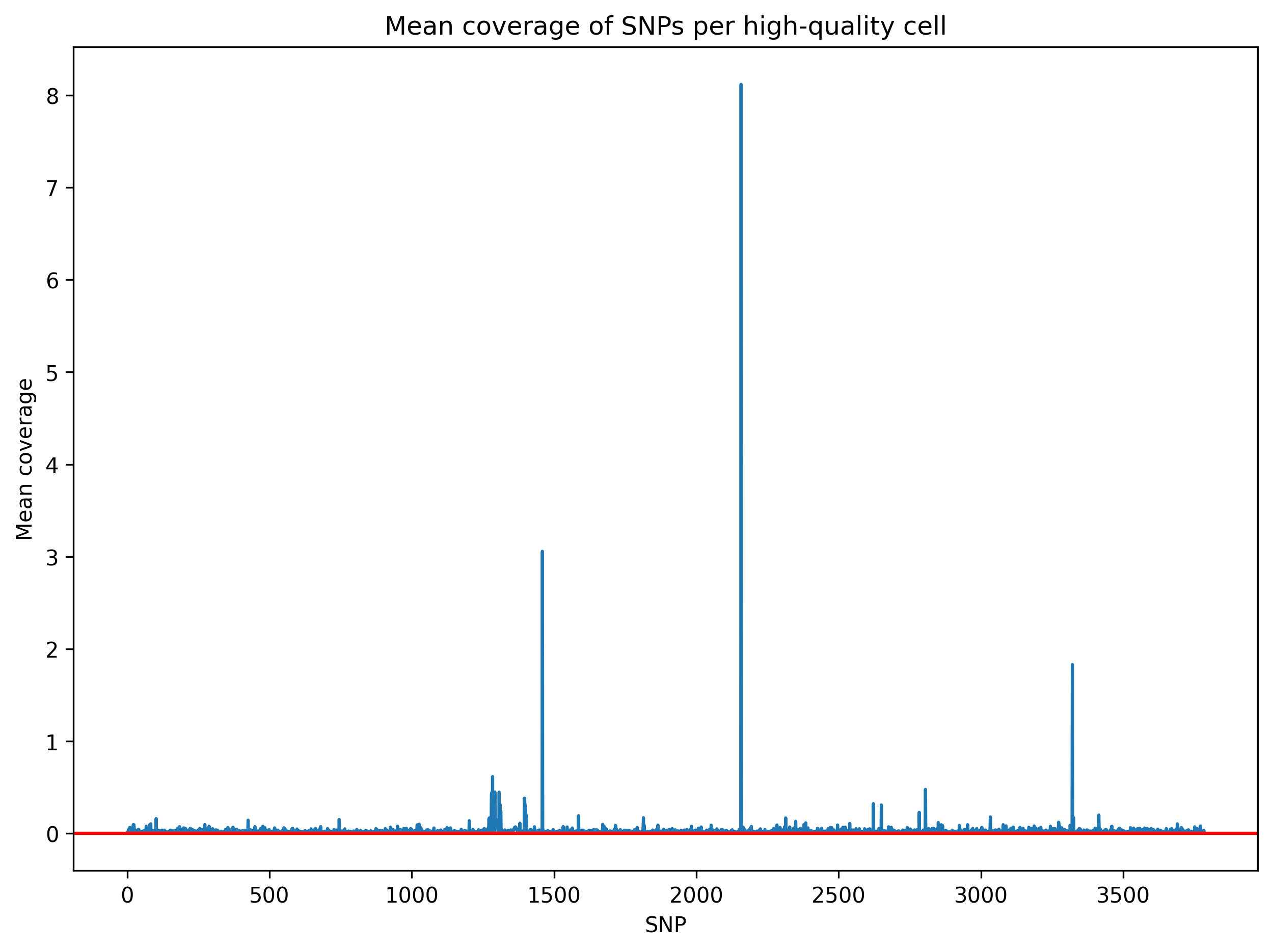

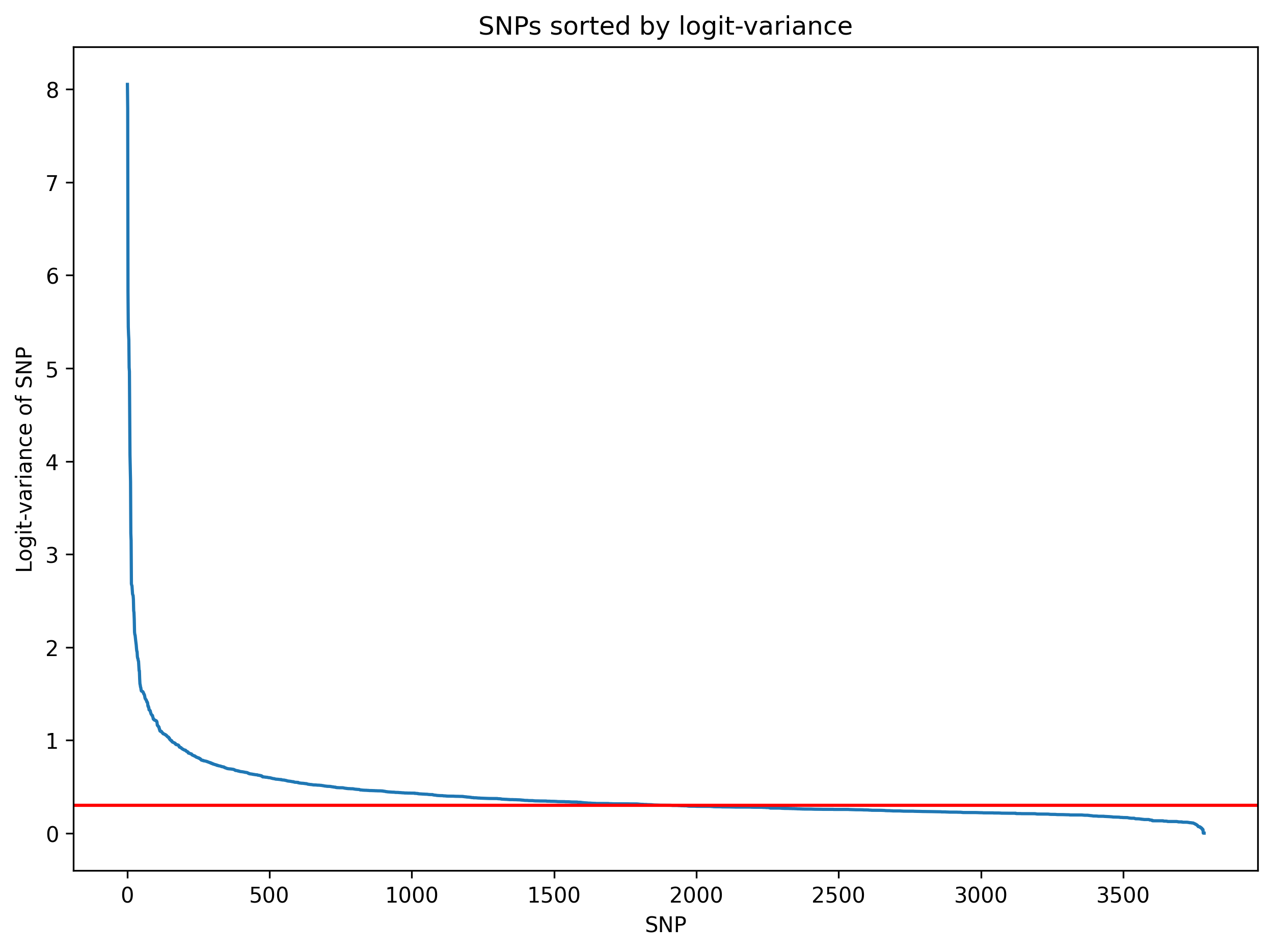


**Supp. Fig. S23.** Pre-filtering of Donor4 dataset. Upper: Number of observed SNPs for each cell. Lower-left: Mean coverage of each SNP. Lower-right: Logit-variance of each SNP. SNPmanifold.filtering() only includes high-quality SNPs and cells higher than the red lines input by users.


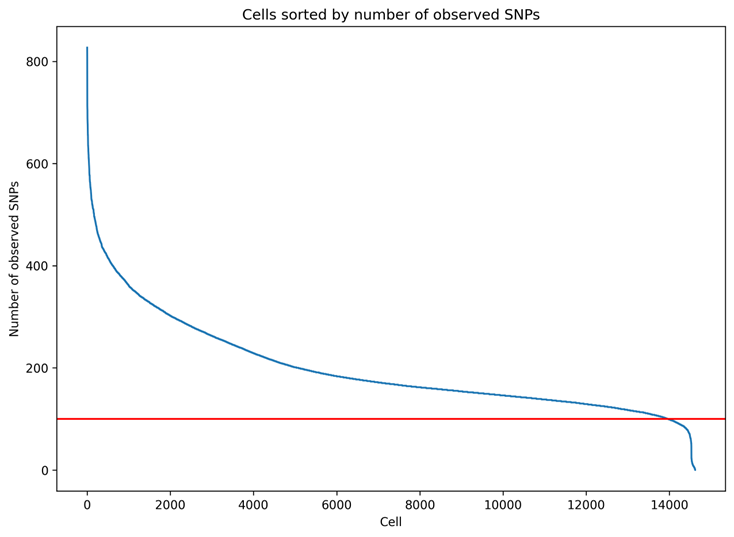

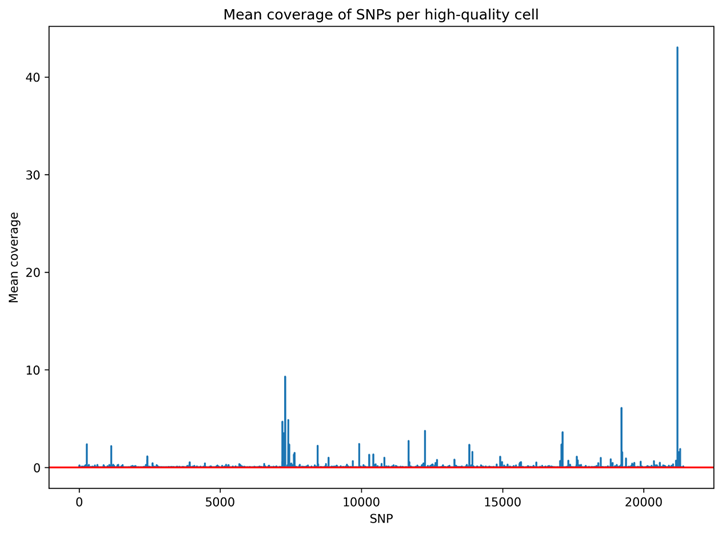

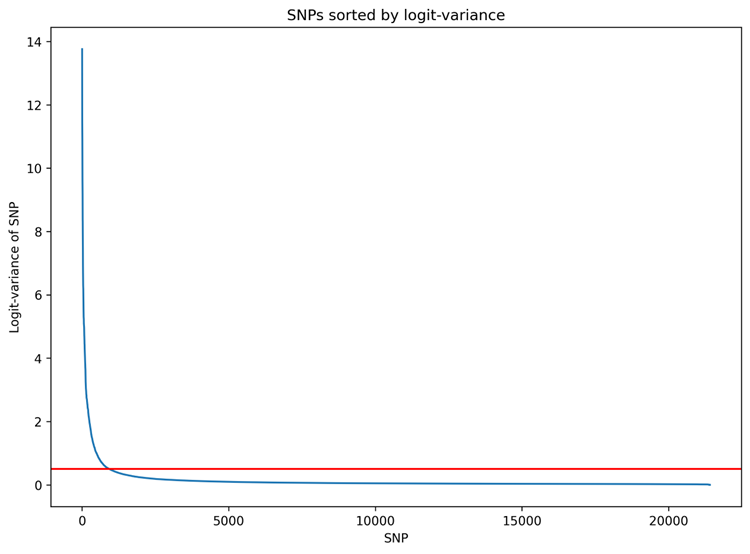


**Supp. Fig. S24.** Pre-filtering of Donor8 dataset. Upper: Number of observed SNPs for each cell. Lower-left: Mean coverage of each SNP. Lower-right: Logit-variance of each SNP. SNPmanifold.filtering() only includes high-quality SNPs and cells higher than the red lines input by users.


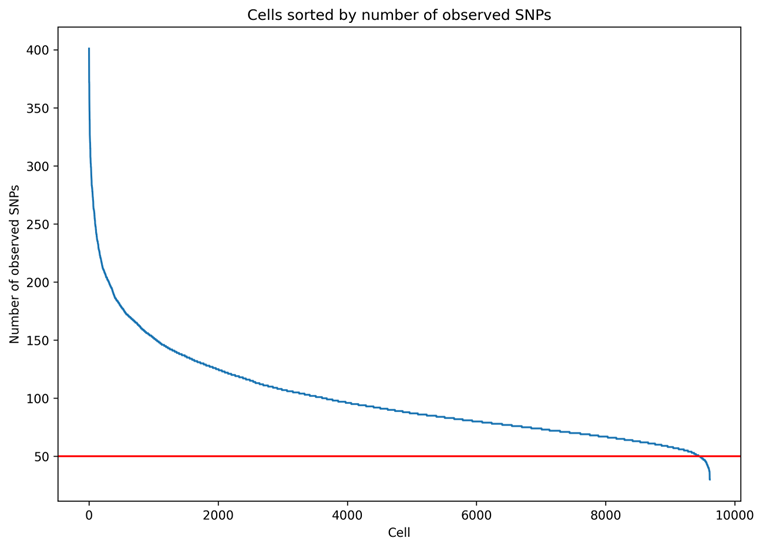

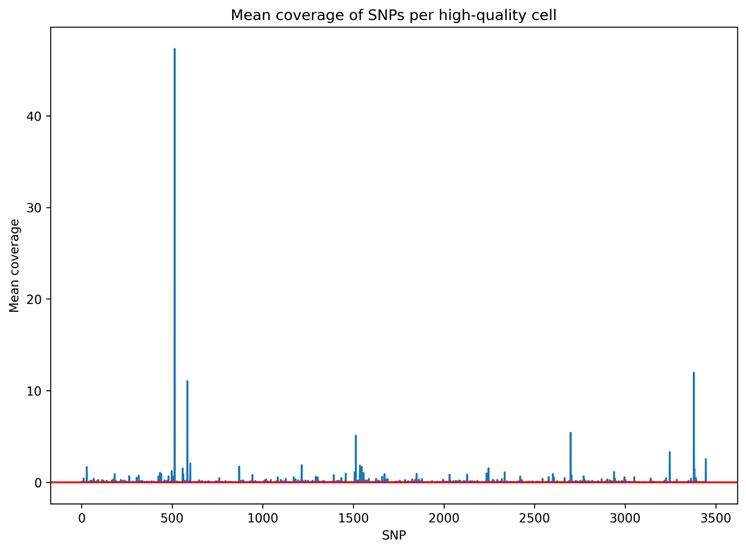

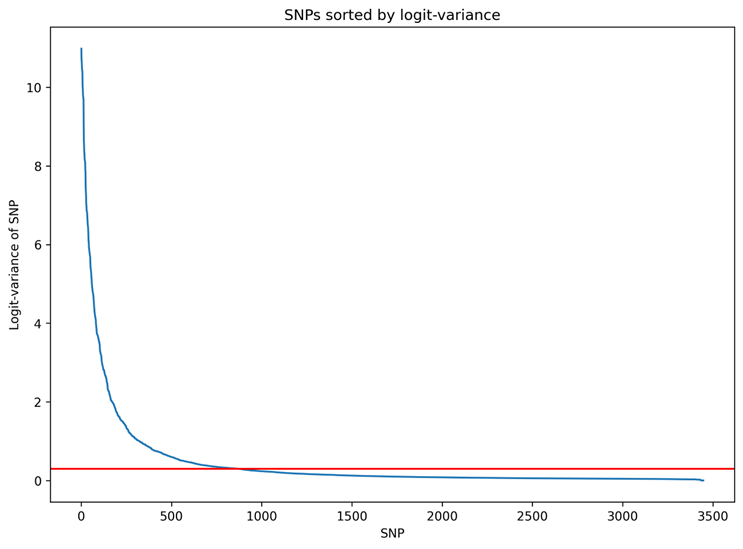


**Supp. Fig. S25.** Pre-filtering of Donor18 dataset. Upper: Number of observed SNPs for each cell. Lower-left: Mean coverage of each SNP. Lower-right: Logit-variance of each SNP. SNPmanifold.filtering() only includes high-quality SNPs and cells higher than the red lines input by users.


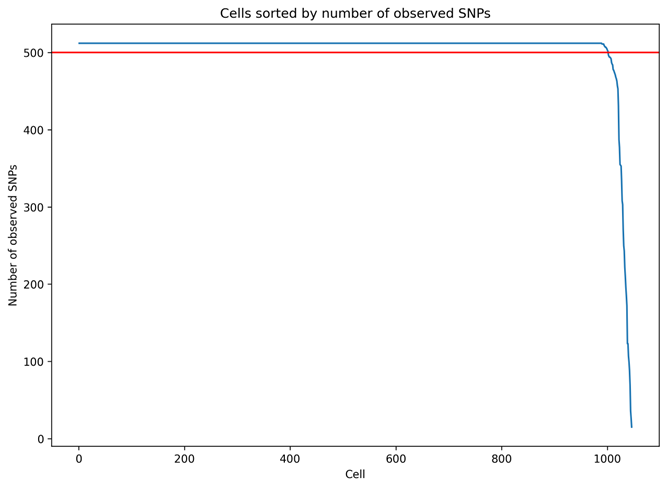

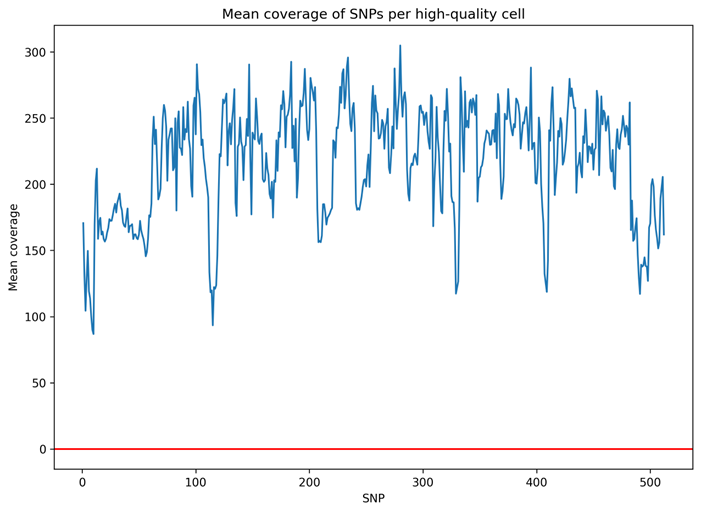

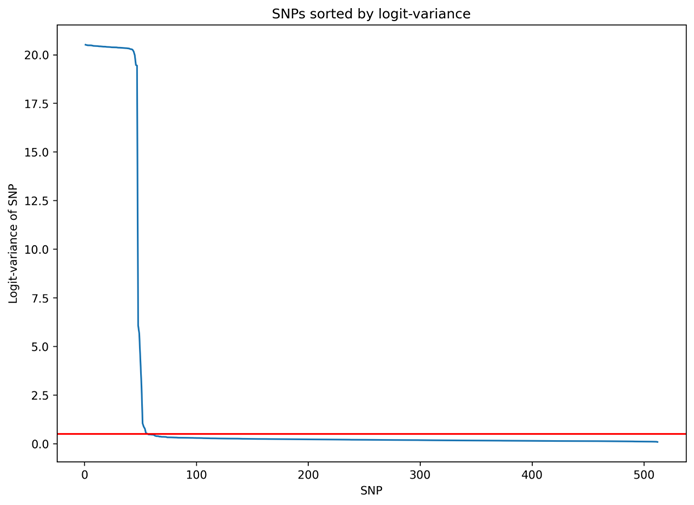


**Supp. Fig. S26.** Pre-filtering of TF1_GM11906 dataset. Upper: Number of observed SNPs for each cell. Lower-left: Mean coverage of each SNP. Lower-right: Logit-variance of each SNP. SNPmanifold.filtering() only includes high-quality SNPs and cells higher than the red lines input by users.


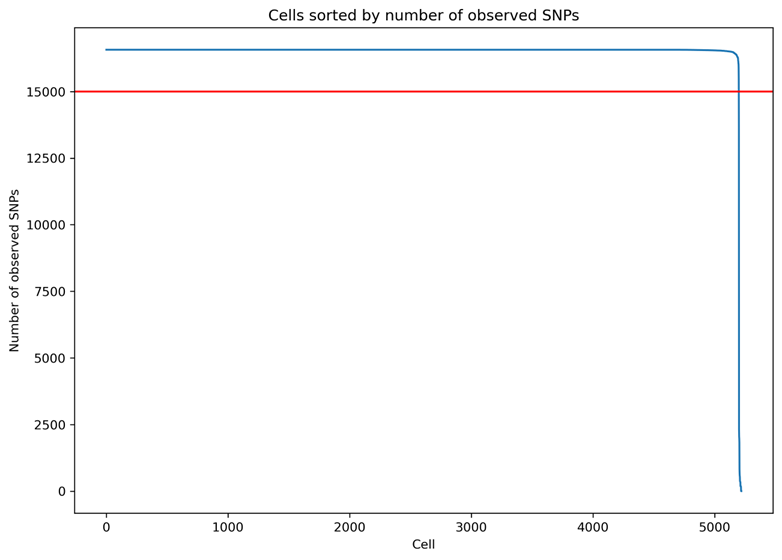

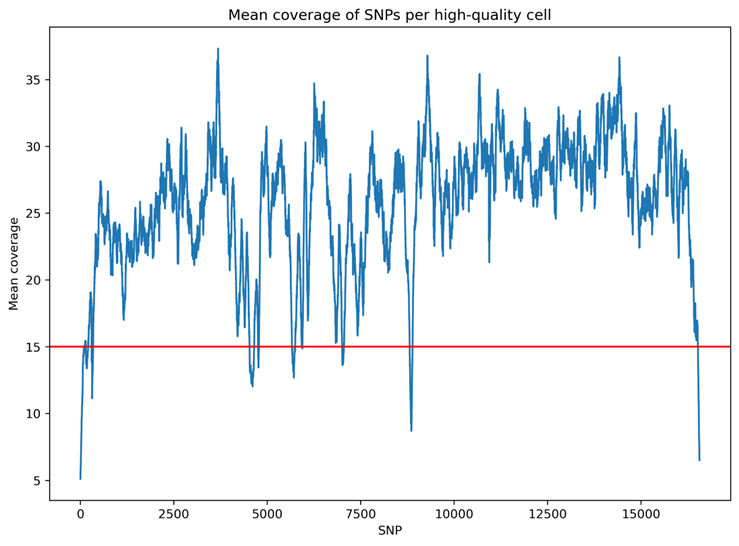

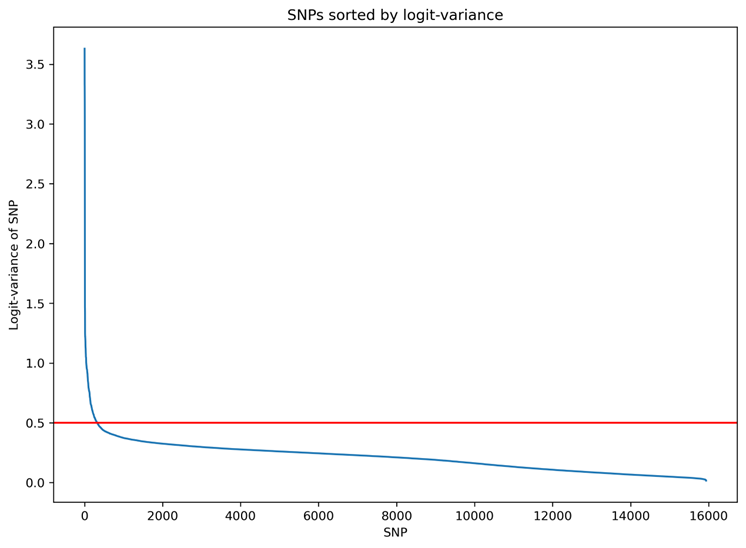


**Supp. Fig. S27.** Pre-filtering of MKN45 dataset. Upper: Number of observed SNPs for each cell. Lower-left: Mean coverage of each SNP. Lower-right: Logit-variance of each SNP. SNPmanifold.filtering() only includes high-quality SNPs and cells higher than the red lines input by users.


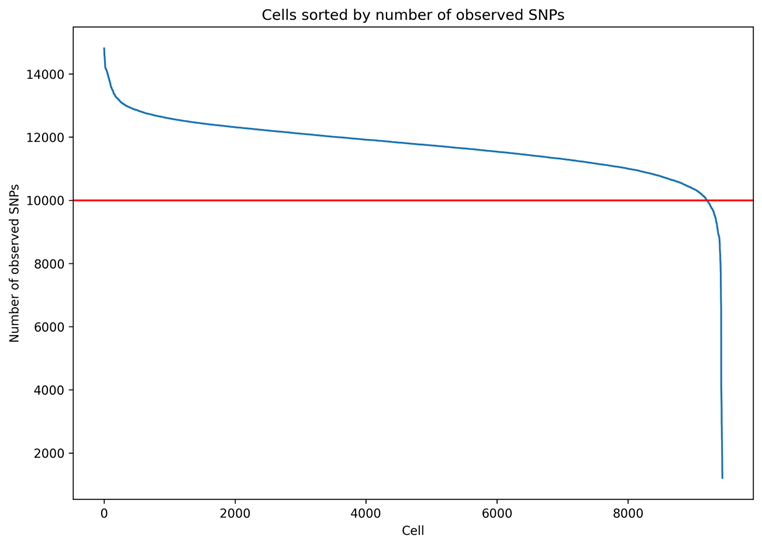

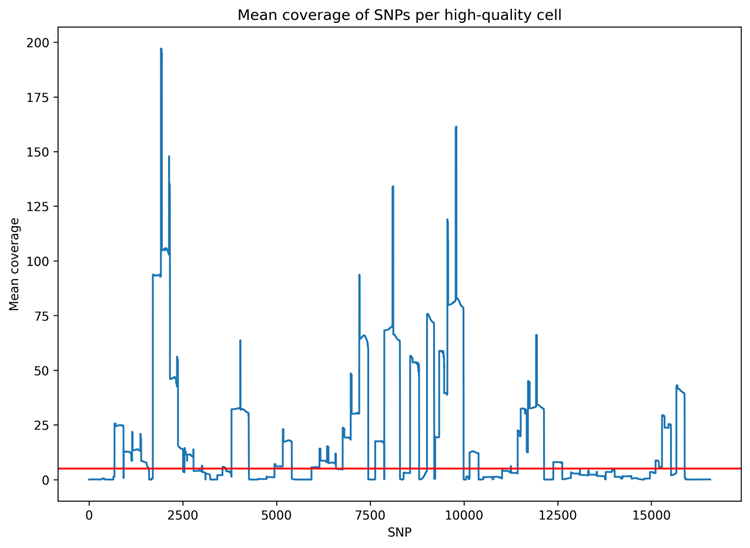

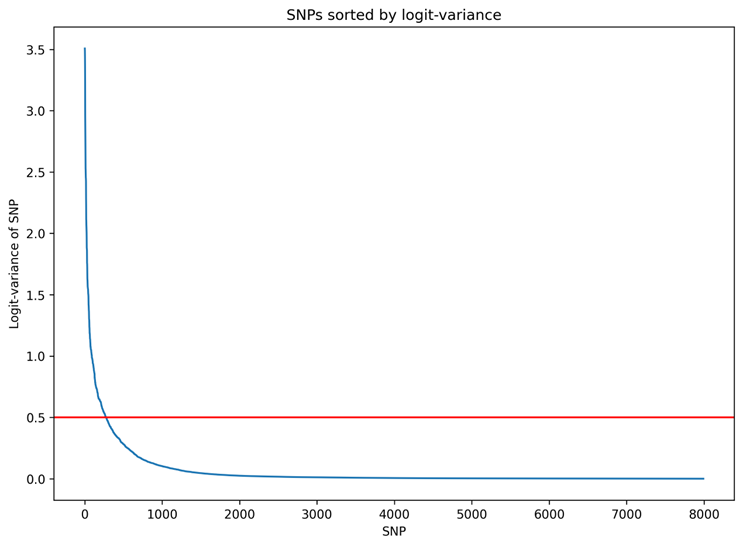


**Supp. Fig. S28.** Pre-filtering of BPDCN dataset. Upper: Number of observed SNPs for each cell. Lower-left: Mean coverage of each SNP. Lower-right: Logit-variance of each SNP. SNPmanifold.filtering() only includes high-quality SNPs and cells higher than the red lines input by users.


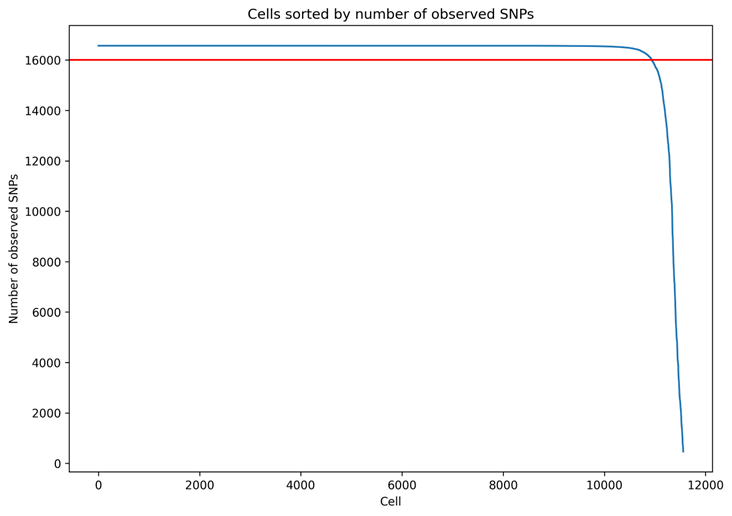

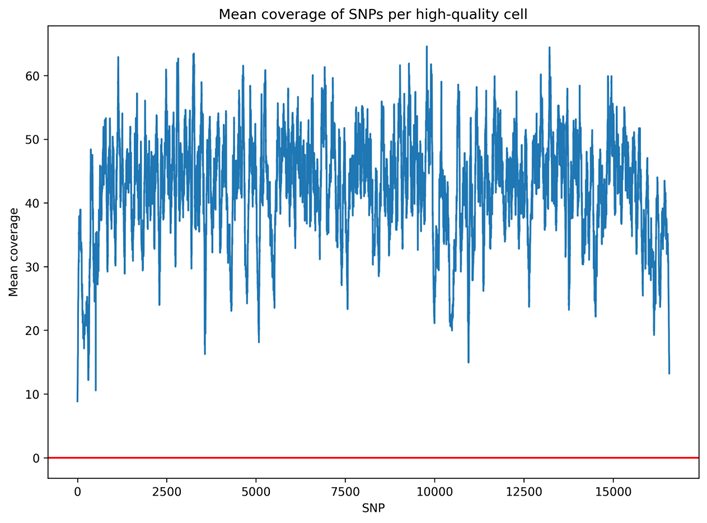

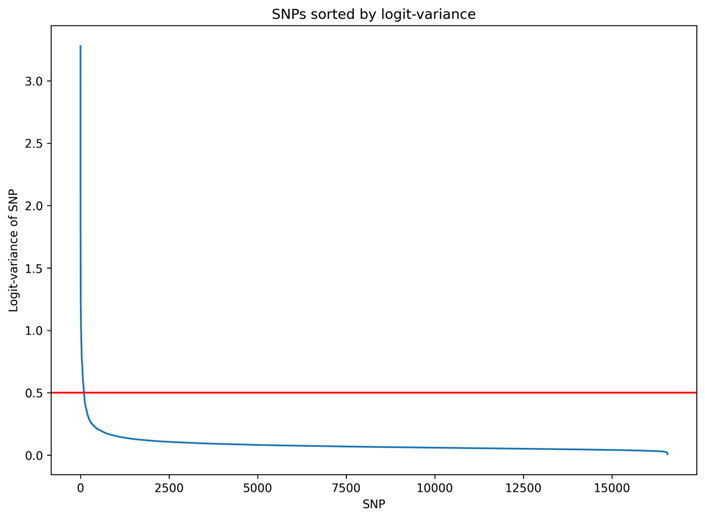


**Supp. Fig. S29.** Pre-filtering of HSPC_PBMC dataset. Upper: Number of observed SNPs for each cell. Lower-left: Mean coverage of each SNP. Lower-right: Logit-variance of each SNP. SNPmanifold.filtering() only includes high-quality SNPs and cells higher than the red lines input by users.
